# A multi-omic *Nicotiana benthamiana* resource for fundamental research and biotechnology

**DOI:** 10.1038/s41477-023-01489-8

**Published:** 2023-08-10

**Authors:** Buddhini Ranawaka, Jiyuan An, Michał T. Lorenc, Hyungtaek Jung, Maria Sulli, Giuseppe Aprea, Sally Roden, Victor Llaca, Satomi Hayashi, Leila Asadyar, Zacharie LeBlanc, Zuba Ahmed, Fatima Naim, Samanta Bolzan de Campos, Tal Cooper, Felipe F. de Felippes, Pengfei Dong, Silin Zhong, Victor Garcia-Carpintero, Diego Orzaez, Kevin J. Dudley, Aureliano Bombarely, Julia Bally, Christopher Winefield, Giovanni Giuliano, Peter M. Waterhouse

**Affiliations:** 1 Centre for Agriculture and the Bioeconomy, Queensland University of Technology (QUT), Brisbane, Queensland Australia; 2 ARC Centre of Excellence for Plant Success in Nature & Agriculture, Brisbane, Queensland Australia; 3 Italian National Agency for New Technologies, Energy and Sustainable Economic Development (ENEA), Casaccia Research Centre, Rome, Italy; 4 Genomics Technologies, Corteva Agriscience, Johnston, IA USA; 5 State Key Laboratory of Agrobiotechnology, School of Life Sciences, The Chinese University of Hong Kong, Hong Kong, China; 6 Instituto de Biología Molecular y Celular de Plantas (IBMCP), Consejo Superior de Investigaciones Científicas (CSIC), Universidad Politècnica de Valencia, Valencia, Spain; 7 School of Biology and Environmental Science, Queensland University of Technology (QUT), Brisbane, Queensland Australia; 8 QUT Central Analytical Research Facility, Queensland University of Technology (QUT), Brisbane, Queensland Australia; 9 Università degli Studi di Milano, Milan, Italy; 10 Department of Wine Food and Molecular Biosciences, Lincoln University, Lincoln, New Zealand; 11 Present Address: Centre for Animal Science, Queensland Alliance for Agriculture and Food Innovation (QAAFI), The University of Queensland, Brisbane, Queensland Australia; 12 Present Address: Centre for Crop and Disease Management, School of Molecular and Life Sciences, Curtin University, Bentley, Western Australia Australia

**Keywords:** Epigenomics, Genome evolution, Comparative genomics, Mobile elements

## Abstract

*Nicotiana benthamiana* is an invaluable model plant and biotechnology platform with a ~3 Gb allotetraploid genome. To further improve its usefulness and versatility, we have produced high-quality chromosome-level genome assemblies, coupled with transcriptome, epigenome, microRNA and transposable element datasets, for the ubiquitously used LAB strain and a related wild accession, QLD. In addition, single nucleotide polymorphism maps have been produced for a further two laboratory strains and four wild accessions. Despite the loss of five chromosomes from the ancestral tetraploid, expansion of intergenic regions, widespread segmental allopolyploidy, advanced diploidization and evidence of recent bursts of Copia pseudovirus (Copia) mobility not seen in other *Nicotiana* genomes, the two subgenomes of *N. benthamiana* show large regions of synteny across the Solanaceae. LAB and QLD have many genetic, metabolic and phenotypic differences, including disparate RNA interference responses, but are highly interfertile and amenable to genome editing and both transient and stable transformation. The LAB/QLD combination has the potential to be as useful as the Columbia-0/Landsberg errecta partnership, utilized from the early pioneering days of *Arabidopsis* genomics to today.

## Abstract (web summary)

Chromosome-level genome reference sequence assemblies of the model and biofactory *Nicotiana benthamiana* line, and a wild relative, have been generated and annotated for gene models, tissue-specific transcriptomes, microRNAs and epigenetic landscapes.

## Main

**Fig. 1:**
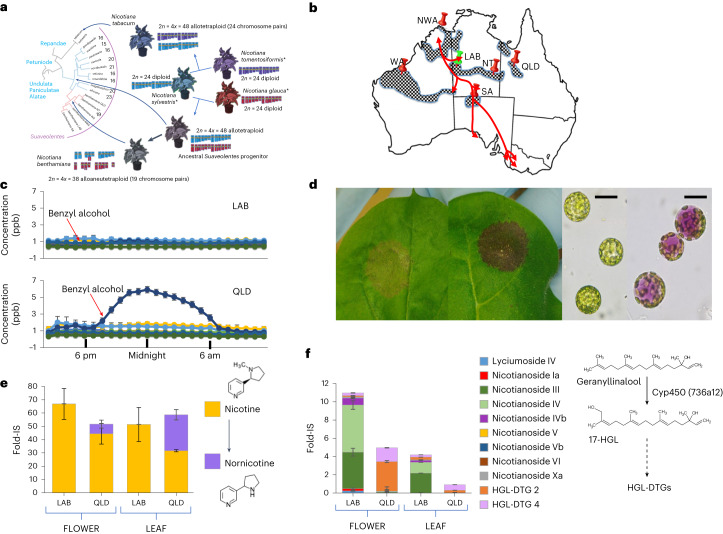
Phenotypic and biochemical diversity of *N. benthamiana*. **a**, Proposed phylogeny and origin of the *Suaveolentes* section compared with other *Nicotiana*s. Chromosome numbers are indicated for each *Suaveolentes* species. Species highlighted by an asterisk are extant relatives of the putative parents of *N. benthamiana* and *N. tabacum*. **b**, Distribution of *N. benthamiana* in Australia (chequered regions). The physical locations of isolated *N. benthamiana* accessions reported in this study are shown by pins, and traditional indigenous trading routes are shown by red lines. **c**, Profiles of average emission of selected floral volatile compounds from LAB and QLD over a 24-h period. Dark blue, benzyl alcohol. For other compounds see Extended Data Fig. 1. Data are presented as mean ± s.e.m. (*n* = 4 per sample point). **d**, Anthocyanin production 5 days after transient expression of AN-like MYB in LAB and QLD; right-hand panels show protoplasts isolated from LAB and QLD infiltrated patches (*n* = 5). Scale bar, 50 μm. **e**, Comparison of the accumulation of nicotine and nornicotine in flowers and leaves of LAB and QLD. The biochemical conversion of nicotine to nornicotine, mediated by the CYP82E demethylase (Extended Data Fig. 9), is shown on the right. Data are presented as mean ± s.e.m. (*n* = 4). **f**, Comparison of the accumulation of HGL-DTGs in flowers and leaves of LAB and QLD. The schematic biochemical pathway is shown on the right. Data are presented as mean ± s.d. (*n* = 4).

The genus *Nicotiana*, comprising ~75 species, is predominantly endemic to the Americas and Australia^1^. Like most Solanaceae, it has a basic chromosome number of 12, with haploid DNA content ranging from 1.37 to 6.27 Gb (ref. ^2^). Section *Suaveolentes* (nicely smelling) includes *N. benthamiana* and is the largest allotetraploid group in the genus (~35 species) with chromosome numbers ranging from 15 to 24, diagnostic of an allotetraplodization event followed by chromosome loss^3–5^ (Fig. 1a). Almost all species in this section are indigenous to Australasia, which they apparently colonized during the Pliocene transition ~5–6 million years ago (Ma). The diploid ancestors of *N. benthamiana* most likely belonged to the *Sylvestres* and *Noctiflorae* sections, whose closest sequenced extant relatives are *N. sylvestris* (~2.6 Gb) and *N. glauca* (~3.2 Gb)^6–11^, respectively.

*N. benthamiana* is a very important plant platform for biopharmaceutical protein and vaccine production^7,12^ and has been instrumental for fundamental discoveries in RNA interference (RNAi), plant–pathogen interactions, metabolic pathway engineering, functional genomics, synthetic biology and gene editing^13^. All this work has relied on plants derived from one accession that we term LAB, which appears to have originated from a single collection near the Granites gold mine in central Australia^7,14,15^ (Fig. 1b). Several additional accessions have recently been described^7,14–16^.

In this paper, we report whole-genome, epigenome and metabolome information for the LAB strain and the wild QLD accession, coupled with single nucleotide polymorphism (SNP) maps for further laboratory and wild accessions. We examine their relationships across the Solanaceae and seek to understand both the evolutionary forces at play and the basis of LAB’s amenability as a research tool.

## Results

### Additional *N. benthamiana* accession resource

**Extended Data Fig. 1:**
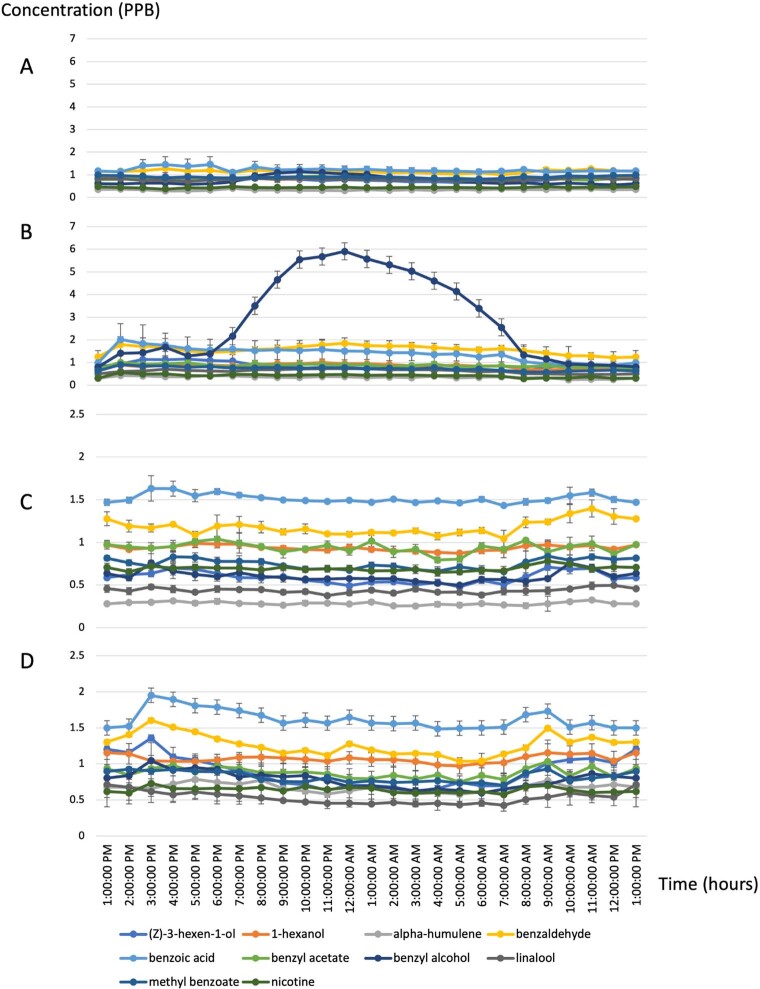
Profiles of average emission of selected putative insect-attracting volatile compounds and nicotine. Profiles of average emission of selected putative insect-attracting volatile compounds and nicotine (a defence compound) in green leaf and floral headspace of LAB and QLD over a 24-hr period. (**A**) LAB floral headspace (**B**) QLD floral headspace (**C**) LAB green leaf headspace (**D**) QLD green leaf headspace. These results indicate that QLD flowers, but not LAB flowers or LAB and QLD leaves, emit benzyl alcohol overnight (6:00 pm–8:00 am). Error bars represent the standard error of the mean (n = 4 per sample point).

**Extended Data Fig. 2:**
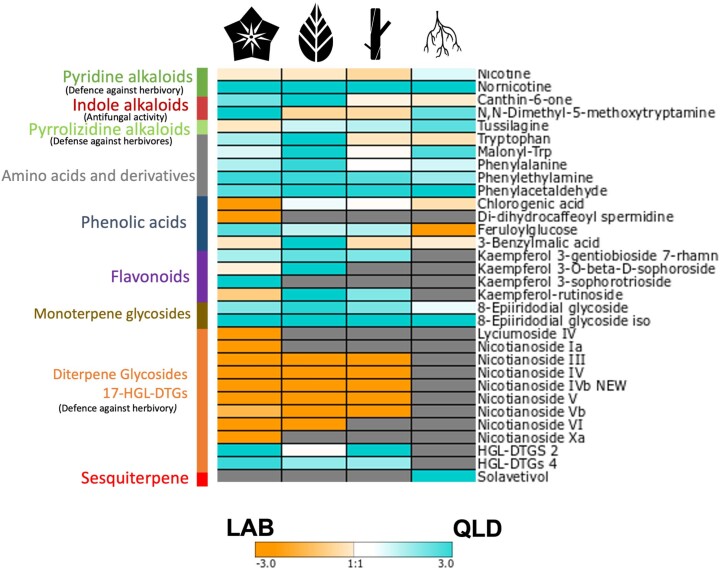
Differentially accumulated metabolites in semi-polar extracts of tissues from *N. benthamiana* LAB and QLD. Differentially accumulated metabolites in semi-polar extracts of *N. benthamiana* LAB vs QLD tissues analysed by liquid chromatography/high resolution mass spectrometry (LC/HESI/MS). The degree of orange/blue indicates relative levels in LAB vs QLD, grey shaded areas not detectable levels.

**Extended Data Fig. 3:**
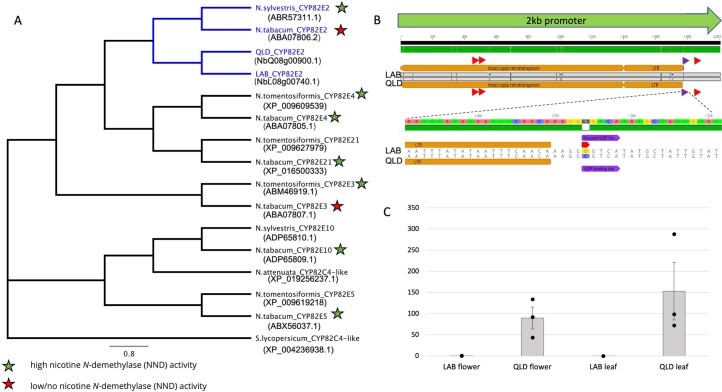
Cladogram of relationships of the nicotine demethylase genes in *S. lycopersicum N. sylvestris, N. tabacum, N. tomentosiformis, N. attenuata*, and *N. benthamiana* (LAB and QLD). Cladogram of relationships of the nicotine demethylase genes in *S. lycopersicum, N. sylvestris, N. tabacum, N. tomentosiformis, N. attenuata*, and *N. benthamiana* (LAB and QLD). The highlighted clade contains the *N. benthamiana* CYP82E2 gene. Genes without stars represent proteins of uncharacterized nicotine N-demethylase activity. (B) Location of bZIP transcription factor binding motifs (red and purple triangles) in LAB and QLD 2kb promoter. The bottom panel shows the transversion in the third TF binding motif (purple triangles) that probably inhibits TF binding and expression of CYP82E2 in LAB. (C) Gene expression (TPM) of CYP82E2 in leaf and flower tissues of LAB and QLD. Error bars represent the standard error of the mean (n=3 biologically independent flower and leaf samples of LAB and QLD).

The QLD wild accession exhibits many morphological, developmental and metabolic differences from LAB^7,14–16^, such as outcrossing flowers, floral scent production at night and the robust capacity to produce anthocyanins (Fig. 1c,d, Extended Data Fig. 1, Supplementary Fig. 1 and Supplementary Table 1). Most notably, QLD is much less susceptible to viruses than LAB, which has been associated with a difference in RNAi competence^7,14^. The levels of a range of metabolites such as phenolic acids, flavonoids, amino acid derivatives and metabolites involved in defence responses^17–20^, such as nornicotine and hydroxygeranyl-linalool diterpene glycosides (HGL-DTG), exhibit marked differences between LAB and QLD (Fig. 1e,f, Extended Data Figs. 2 and 3 and Supplementary Table 2). LAB exhibited a higher number of underexpressed/non-functional biosynthetic pathways than QLD, except for phenolic acids and HGL-DTGs. Because of these and potentially many more differential characteristics, their genetic distance (Fig. 1a) and particularly their differences in viral defence capacity, both LAB and QLD were chosen for chromosome-level genome sequence assemblies.

### Genome assembly, annotation and genetic diversity

**Extended Data Fig. 4:**
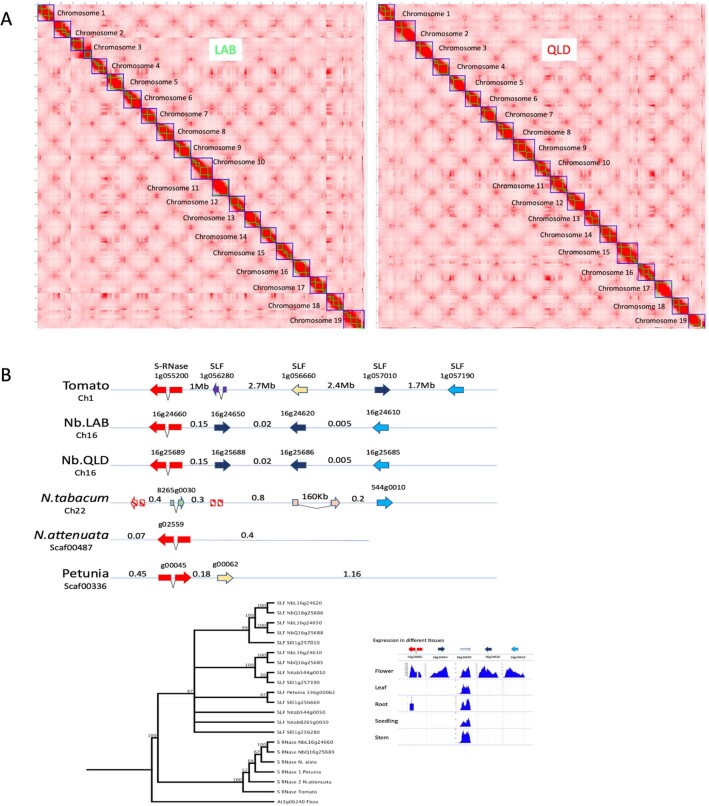
(A).Plot of contact matrices of LAB and QLD assemblies. (B).Synteny of Self-incompatibility (S)-like loci in tomato, *N. attenuata*, *N.tabacum*, petunia, LAB and QLD, cladogram of gene sequence similarities and tissue- expression of mRNA LAB S-locus genes. **(a**) Plot of contact matrices of LAB and QLD assemblies. Juicebox plot from HiC analysis showing resolution into 19 contiguous elements (chromosomes) for both LAB and QLD assemblies. **(b)** Synteny of self-incompatibility (S)-like loci in tomato, *N. attenuata*, *N.tabacum*, petunia, LAB and QLD, cladogram of protein gene sequence similarities and tissue-specific mRNA expression of the LAB S-locus. Gene arrangement and relationships in cartoon form of the genes in the highly recombinogenic S-locus (comprised of an S-RNAse and associated multiple copies of F-box (SLF) proteins) in the most advanced genome assemblies of tomato, *N. attenuata*, *N.tabacum*, petunia, LAB and QLD. The colours of the genes represent their relationships across species, as indicated in the cladogram. The analysis shows contiguity of the S-locus in tomato, LAB and QLD and the fragmented nature of the locus in *N. attenuata*, *Petunia axillaris*, due to their presence on small scaffolds, and the incomplete assembly of Ch22 in *N.tabacum*. Tissue expression data for LAB shows that the intervening gene 16g24630 is expressed in all 5 tissues examined but the S-RNAse and SLF genes are expressed only in the floral tissue, as expected for a floral incompatability-associated locus. Distances between genes are indicated in Mb.

Long and short sequence reads of the LAB and QLD accessions were assembled into 19 chromosomes for each genome (Methods and Supplementary Fig. 2). The chromosomes ranged in size from 128 to 182 Mb, with total genome sizes of ~2.8 Gb (LAB) and ~2.9 Gb (QLD), of which 99% and 96% respectively anchored to chromosomes (Supplementary Table 3). This represents ~94% of the expected genome size estimated from cytological staining^2^. The assemblies were annotated (Methods and Supplementary Fig. 2) to 45,797 and 49,636 gene models in LAB and QLD (Supplementary Table 3) respectively. Approximately 87% of the gene models in LAB and 75% in QLD are fully supported by RNA-sequencing (RNA-seq) (Supplementary Tables 4 and 5) and 98% of LAB expressed sequence tag sequences^21–23^ mapped to the LAB genome coding sequences. According to several quality scores, including the long terminal repeat (LTR) Assembly Index^24^, the LAB and QLD assemblies were well above the standard requirements of the Earth Biogenome Project^25,26^ (Supplementary Table 6). They have higher contiguity than any published *Nicotiana* genome assemblies (Table 1); this is further illustrated by the contact matrices (Extended Data Fig. 4(A)) and analysis of the well-studied *S* locus (Extended Data Fig. 4(B)).

**Table 1:** Genome assembly metrics of LAB and QLD compared with reference genomes

Species/accession	Scaffolds >500 nucleotides	Chromosome	L50	N50 (Mb)	Assembled genome length (Gb)	BUSCO % Complete v10
*N. benthamiana* LAB	19	19	10	145	2.75	C:98.1%[S:46.0%,D:52.1%]
*N. benthamiana* QLD	19	19	10	141	2.72	C:98.0%[S:47.5%,D50.5%]
*Arabidopsis thaliana*	5	5	3	23	0.12	C:99.2%[S:98.7%,D:O.5%]
Potato (dihaploid)	12	12	6	59	0.74	C:98.4%[S:96.6%,D:1.8%]
Tomato	12	12	6	61	0.72	C:97.8[S:96.8%,D:1.0%]
Eggplant	12	12	5	76	0.83	C:84.2%[S:82.7%,D1.5%]
Tobacco chromosomes (scaffolds)	24 (942,183)	24	9 (3,998)	84 (0.22)	1.74 (4.01)	C:82.6%[S:61.2%,D:21.4%] (C:96.8% [S:24.3%, D:72.5%1])
*Capsicum*	12	12	6.00	221	2.56	C:74.8%[S:73.7%,D:1.1%]
*N. attenuata*	12 (37,194)	12	498 (1,627)	66 (0.45)	0.73 (2.09)	C:48.5% [S:47.4%, D:1.1%] (C:98.1% [S:95.9%, D:2.2%])
*Petunia axilaris*	17,630	12	17,630	1.24	1.20	C:98.2%[S:95.6%,D:2.6%]
*N. benthamiana* LAB (USA vI.0.I)	52,890	19	1,718	0.44	2.49	C:98.2%[S:45.8%,D:52.4%]
*Petunia inflata*	35,907	12	35,907	0.88	1.17	C:97.9%[S:91.6%,D:6.3%]
*N. benthamiana* LAB (AU v0.5)	77,255	19	1,903	0.39	2.49	C:97.6%[S:47.5%,D:50.1%]
*N. sylvestris*	125,957	12	7,255	0.08	2.01	C:95.1%[S:93.3%,D:1.8%]
*N. tomentosiformis*	90,682	12	5,563	0.15	1.62	C:94.4%[S:92.6%,D:1.8%]
*N. obtusifolia*	20,758	12	2,189	0.05	3.50	C:94.3%[S:92.3%,D:2.0%]
*N. otophora*	420,947	12	14,141	0.03	2.32	C:76.0%D[S:74.3%,D:1.7%]

Various genome assembly quality criteria (L50, N50, BUSCO score) are used to compare *N. benthamiana* with the other available genomes. The values in parentheses for tobacco and *N. attenuata* are those obtained from scaffold data alone. L50, count of smallest number of sequences whose length sum makes up 50% of the genome assembly.

**Extended Data Fig. 5:**
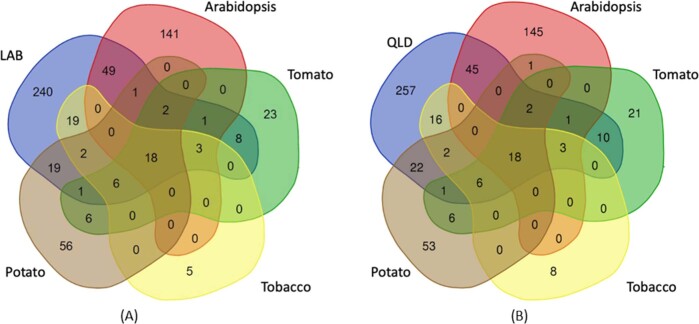
miRNA families in LAB and QLD shared with *A. attenuata*, *S. lycopersicum*, and *S. tuberosum*. The number of identified miRNA families in LAB and QLD that are shared with three Solanaceae plants (*A. attenuata*, *S. lycopersicum*, and *S. tuberosum*) and the well-studied plant Arabidopsis (*A. thaliana*) are illustrated in a Venn diagram. The figure shows that the major miRNAs in the most related plant, *N. attenuata*, were identified in both LAB and QLD. Many potential miRNAs were discovered that have not been previously identified. Subfigure (**a**) shows the overlapping number of identified miRNAs in LAB that are shared with the other four species. Subfigure (**b**) shows the identified miRNAs in QLD.

Gene mapping (Supplementary Table 7a) revealed that 72%, 92% and 89% of the *N. benthamiana* genes are orthologous to those in tomato, *N. attenuata* and tobacco, respectively. Similar numbers were obtained by protein cluster analysis (Supplementary Fig. 3 and Supplementary Table 7b). There were ~1,000 and ~3,000 genes specific to LAB and QLD, respectively. Based on BUSCO scores and comparison of the predicted protein lengths with their *Arabidopsis* best hits, the LAB and QLD annotations are better than most *Nicotiana* and *Solanaceae* annotations (Supplementary Table 7c and Supplementary Fig. 4). A total of 369 and 383 potential microRNA families and the expression of 59 and 57 of them were detected in LAB and QLD, respectively (Supplementary Table 8a–e and Extended Data Fig. 5).

The previously described NT, SA, WA and NWA wild accessions^14^ (Fig. 1b), as well as the extensively used green fluorescent protein (GFP)-expressing transgenic line (16c) produced in D. Baulcombe’s laboratory^23,27^ (EU-LAB) and (USA-LAB) were re-sequenced and mapped onto the LAB and QLD assemblies. SNPs frequencies^28^ (Supplementary Table 9) were very low among the three LAB accessions (<25 SNPs per Mb), showing that our LAB assembly is a tremendous resource for worldwide *N. benthamiana* laboratory isolates; SNPs between the four wild accessions mirrored the previously calculated evolutionary relationships^14^ (Supplementary Table 9) and were similar in range to those of 20 *Capsicum annuum* accessions^29^. SA and LAB, originally collected from geographically well separated locations, have close genetic similarity (~51 SNPs per Mb). One possible explanation is that Pitjuri (a chewing tobacco mixture often containing dried *N. benthamiana* aerial tissue) exchanged along ancient aboriginal traditional trading routes (Fig. 1b) has transported seed between these locations over the past 60,000 years. The annotated genomes of LAB and QLD, containing tracks describing gene models, SNPs with other *N. benthamiana* isolates, gene expression across five tissues, location and expression of pre-miRNAs, and the epigenetic landscapes, are available on an interactive WebApollo browser^30^ (https://www.nbenth.com).

### Homeologous chromosomes, subgenomes and chromosome loss

The genomes of most diploid Solanaceous species consist of 12 chromosome pairs (2*x* = 2*n* = 24) encoding about 35,000 genes^31^. *N. tabacum*, an allotetraploid formed about 0.2–0.4 Ma^8,9^ has 24 chromosome pairs (2*n* = 4*x* = 48) encoding ~70,000 genes^32,33^. In the estimated 5–6 million years since the hybridization event basal to the Australian *Nicotiana* clade, *N. benthamiana* has lost five chromosome pairs to give a genome of 2*n* = 4*x* = 38 (Fig. 1a)^4,5^.

**Fig. 2:**
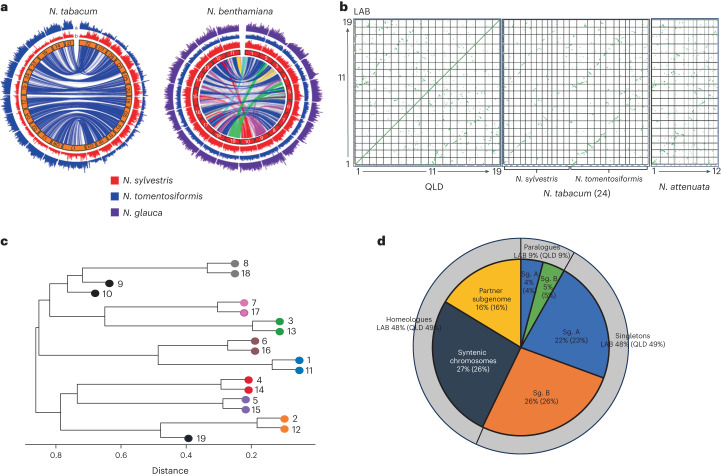
Subgenome and homeologue organization in *N. benthamiana*. **a**, The left-hand Circos plot depicts the locations of the syntenic blocks (1 Mbp) of *N. tomentosiformis* (blue) and *N. sylvestris* (red) on the *N. tabacum* genome, highlighting the subgenomes and their respective contribution to the subgenome structure of this species. The right-hand Circos plot similarly locates the syntenic blocks of *N. tomentosiformis* (blue), *N. sylvestris* (red) and *N. glauca* (purple) on the *N. benthamiana* LAB genome, highlighting the difficulty in assigning ancestry for subgenomes in this species, which is characterized by extensive rearrangement of blocks between individual chromosomes. The lines in the centre join syntenic regions, highlighting the fragmentation of the *N. benthamiana* genome. **b**, Dot plot showing the relationship between the LAB and QLD chromosomes (central continuous line in the far-left panel) and the fragmented syntenic relationship between the subgenomes. Comparison of the *N. tabacum* genome consisting of two subgenomes with clear relationships to *N. sylvestris* and *N. tomentosiform*is revealed a fragmented relationship with *N. benthamiana* chromosomes. **c**, Dendrogram highlighting the chromosome pairs and the three orphan chromosomes (annotated 9, 10 and 19). **d**, Retention and relocation of homeologous genes in *N. benthamiana* LAB and QLD genomes. Percentage values outside and within parentheses are those for LAB and QLD, respectively, and show that about half of the original homeologous pairs have lost one member.

A mapping approach, similar to that used to identify the subgenomic memberships of the *N. tabacum* chromosomes^32–34^, was applied to *N. benthamiana* and *N. tabacum* using sequences from the genomes of *N. sylvestris*, *N. glauca* and *N. tomentosiformis*. This recapitulated the previous tobacco results but, as previously predicted^8,9^, did not differentiate the *N. benthamiana* chromosomes into a *N. glauca*- and a *N. sylvestris*-related subgenome (Fig. 2a). Therefore, we took a different approach. Syntenic sequences and blocks of orthologous genes were compared both within the highly syntenic LAB and QLD genomes and with *N. tabacum*^32^ and *N. attenuata* genome assemblies^34^ (Fig. 2b). A dendrogram, derived from matrices of degrees of similarity of counterpart gene sequences of the *Nicotiana* set, clearly identified eight homeologous chromosome pairs and three orphan chromosomes (Fig. 2c and Supplementary Table 10).

**Fig. 3:**
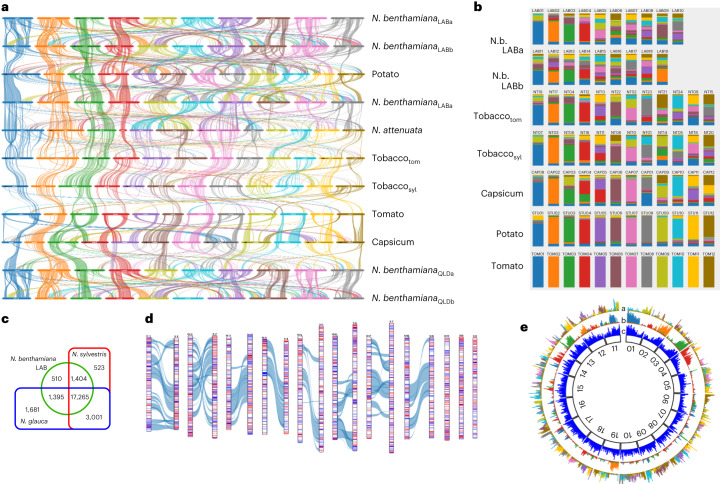
Gene block conservation across the Solanaceae and segmental allopolyploidization in *N. benthamiana*. **a**, Waterfall plot showing the syntenic relationships between LAB, QLD and other related species as generated by SynVisio. **b**, Fraction of orthologous gene clusters in different Solanaceae chromosomes, highlighting a high conservation of chromosomes 1–4, and a declining conservation of remaining chromosomes; chromosome numbering largely follows the tomato–potato system. N.b., *N. benthamiana*. **c**, A Gibson Venn diagram showing the number of gene family clusters that are shared among LAB, *N. sylvestris* and *N. glauca*. **d**, Overlay of *N. glauca* (blue bars within chromosomes) and *N. sylvestris* (red) orthologous genes on LAB chromosomes. Grey/blue lines connecting chromosomes link syntenic blocks among the matched subgenome chromosomes. **e**, Circos plot of the physical distribution of syntenic blocks of tomato chromosomes 9–12 overlaid onto the LAB genome (track a), showing extensive fragmentation across the remaining LAB chromosomes. By contrast, an overlay of the syntenic blocks of tomato chromosomes 1–4 onto the LAB genome clearly demonstrates the conservation of both sequence and location (track b). Track c shows the gene density across the LAB chromosomes.

To separate the genome into two functional subgenomes we took a disjoint subset partitioning approach, enabled by the ~50% of genes for which homeologous gene pairs were identified to be on chromosomes other than their predicted homeologous counterpart. Every combination of LAB chromosomes was assigned to two disjoint subsets and measured for the number of homeologous gene pairs distributed 1:1 between the two subsets. The best combination, excluding the genes on the three orphan chromosomes, gave a distribution of 8,543 gene pairs in opposite subgenomes and 1,999 gene pairs in the same subgenome (Supplementary Table 11a–h and Fig. 2d). Visual comparison of *N. benthamiana* subgenomes with genomes of six other Solanaceous species using SynVisio^35^ revealed remarkable long range synteny across the family, which was even more apparent as the percentage of genes on each chromosome of the species that are orthologous to those on each tomato chromosome, especially in chromosomes 1, 2, 3 and 4, but still discernible in *N. tabacum* up to chromosome 7 (Fig. 3a,b). By contrast, in *N. benthamiana* this conservation declines rapidly after chromosome 4 (Fig. 3b,e), probably because of the high degree of chromosomal rearrangements specific to this allopolyploid species.

The blocks of synteny between the two subgenomes of *N. benthamiana* are more numerous, larger and contiguous than with the *N. sylvestris-*derived subgenome of *N. tabacum* (Supplementary Fig. 5). To investigate this further, a cluster analysis was made using the proteomes predicted from our LAB assembly and the available scaffold assemblies of *N. sylvestris* and *N. glauca* (Fig. 3c). The LAB genes identified as clustering with *N. sylvestris* but not *N. glauca* genes, and vice versa, were mapped onto the LAB genome (Fig. 3d). This revealed that, even in the gene-rich, large, Solanaceae-wide syntenic blocks, extensive recombination has occurred between the two ancestral subgenomes and suggests that the current *N. benthamiana* genome is the result of extensive ‘duplication/deletion’ homeologous recombination^36^, or of repeated hybridization among the derivative populations from the original allotetraploid *Nicotiana* at the base of the *Suaveolentes*. These processes have produced chromosomes composed of genes from both ancestral parents, explaining the greater synteny between *N. benthamiana’s* homeologous chromosomes compared with their *N. sylvestris* counterparts. This is also the probable cause of the low level of subgenome dominance (Supplementary Fig. 6 and Supplementary Table 12). Subgenomes A and B encode 23,408 and 22,388 genes, respectively, and the overall transcript abundance of homeologues differs by only 1%, suggesting that the genome is in balanced but fluid harmony.

### LAB and QLD as model plants and biofactory platforms

**Fig. 4:**
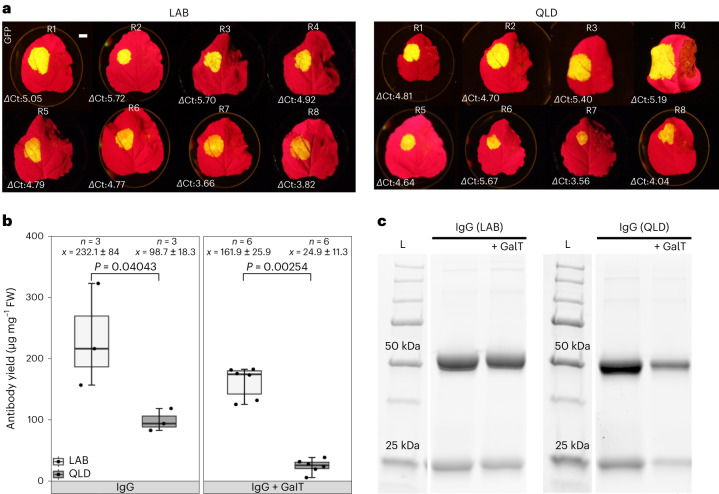
Comparison of transient expression in LAB and QLD of GFP by syringe agro-infiltration and antibody production by vacuum agro-infiltration. **a**, Transient expression of GFP in LAB and QLD. Quantitative polymerase chain reaction cycle threshold (Ct) values were measured and *Δ*Ct calculated as the difference in Ct between the gene of interest (GFP) and the reference gene (GAPDH) for each sample. GFP expression levels are represented underneath each leaf as *Δ*Ct. All reactions were performed in triplicate for each complementary DNA sample. All experiments were performed in eight independent biological replicates. The average *Δ*Ct of LAB and QLD was 4.8 and 4.7, respectively. Statistical analysis of the two-tailed Student’s *t*-test (*P* = 0.7972) and *z*-test (*P* = 0.9949) shows that there was no significant difference between GFP expression levels in the two ecotypes. Scale bar, 1 cm. **b**, Antibody concentration in total soluble protein extracts from LAB (white) and QLD (grey) ecotypes measured by protein A biolayer interferometry in μg mg^−1^ of tissue fresh weight (FW). *P* values were determined by Mann–Whitney *U*-test comparing between ecotypes. For ‘*n*’, samples are biologically independent transient infiltrations, sampled at 7 days post infiltration. Box and whisker plot interpretation: each box spans the interquartile range with the ends of the box being the upper and lower quartiles. The median is represented by a vertical line inside the box. Whiskers outside the box extend to the highest and lowest observations. GalT, galactosyl transferase; IgG, immunoglobulin G. **c**, SDS–polyacrylamide gel electrophoresis showing protein A-purified trastuzumab under reducing condition, similar results were observed in three independent replicates (*n* = 3). Source data

**Extended Data Fig. 6:**
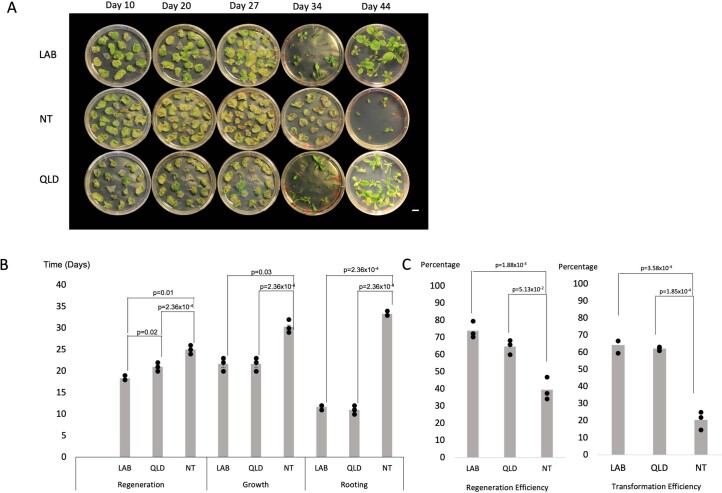
Transformation efficiencies of  LAB, QLD and Northern Territory (NT) accessions. Comparison of transformation efficiencies of LAB, QLD and Northern Territory (NT) accessions. (**a)** Regeneration, selection, shoot development, and root development of LAB, NT and QLD ecotypes post-transformation with a 35S:Cas9 cassette and kanamycin selectable marker (scale bar represents 1 cm). The dates on top of the image indicate the progression of transformation. (**b**) Comparison of time taken for regeneration, growth (1-2 cm shoots) and rooting of LAB, QLD and NT. ANOVA two-tailed test was performed to determine the significance differences. (Data are presented as mean values +/− standard error (n=3 biologically independent samples). (**C**) Comparison of regeneration frequency and transformation efficiency of LAB, QLD and NT. ANOVA two-tailed test without transformation was performed to determine the significance differences between percentage data derived from count data. Independent positive transformants of LAB n=72, QLD n=74 and NT n=21 (a single sister plant derived from one single callus) were used to calculate the transformation efficiency.

**Extended Data Fig. 7:**
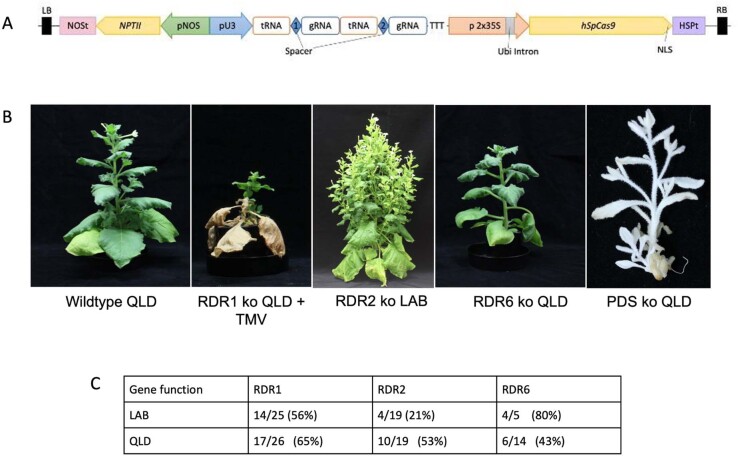
Comparison of CRISPR/Cas9 editing efficiency in LAB and QLD. (**a**) The basic editing construct (with kanamycin selection) used to transform LAB or QLD tissues. The two guide (g)RNA sequences were placed between the tRNA processing units (indicated as spacer sequences 1 & 2 in panel A). Two sites were chosen within the same target gene, usually ~200 nucleotides apart, and gave either a dropout of the intervening DNA sequence in the genome or inaccurate repair of one or both sites. (**b**) Phenotypes of QLD knockouts (ko) of RDR1 infected with Tobacco mosaic virus (TMV), RDR6 and Phytoene desaturase (PDS) and LAB knockout of RDR2. Silencing of PDS in QLD targeted two homoeologs simultaneously to give biallelic silencing of both genes in the T0 generation. gRNA sequences used: *RNA-dependent RNA polymerase* (NbRDR1): TAAATAGTACAGTTTCTCCA; GACACTCAAAGTTTCTCTGG. NbRDR2: CCACTCCCAACGTAGATAAG; GTGTCTCGAAATGTGCTGCA. NbRDR6: CTTACTTAGAAGTCATCAGG; CTGCAACAGTATTACCAAAG. *Phytoene desaturase* (NbPDS) TCACAAACCGATATTGCTGG; GAGCTTCAGGAAAATCAAAG. (**c**) Comparison of editing efficiency of LAB and QLD. Editing efficiency in LAB and QLD was determined using the NbRDR genes involved in RNAi.

An impaired RNAi response in *N. benthamiana*–LAB may underlie the plant’s excellence as a biofactory and research tool^7^. To examine this, the capacity for transgenesis, genome editing, transient transgene expression and the presence, integrity and expression levels of RNAi-associated genes were analysed in LAB and QLD (Supplementary Fig. 7). In both accessions, principal viral defence RNAi genes^37^, *DCL2*, *RDR6*, *DRB4* and *AGO2* have one expressed homeologue, both functional *DCL4* homeologues and four expressed copies of *AGO1*. The number, integrity and expression of these genes does not differ significantly between the accessions, nor does those of RNAi genes involved in chromatin remodelling or endogenous small RNA production (Supplementary Fig. 7). *NbRDR1* is the exception. In LAB, there is a 72 nucleotide insertion that creates stop codons towards the middle of the gene^38^. Curiously, the messenger RNA is full length and accumulates like that of its uninterrupted QLD counterpart. Nonetheless, the truncated NbRDR1 protein in LAB is not acting as a dominant negative because engineering early stop codons into the gene did not relieve the viral susceptibility (Supplementary Fig. 8). To test whether the difference in RDR1 function might make QLD a superior or inferior research tool and bioplatform to LAB, the accessions were assessed for ease and efficiency of transformation, and gene editing and level of transient gene expression from syringe and vacuum infiltration (Extended Data Figs. 6 and 7, Supplementary Table 13 and Fig. 4). In almost all of these respects they performed similarly. However, LAB yielded a much higher level of transiently expressed antibody from vacuum agro-infiltration (Fig. 4b,c), is physically easier to patch-infiltrate and has a faster generation time^14^.

### Expansion and contraction of transposable elements

**Fig. 5:**
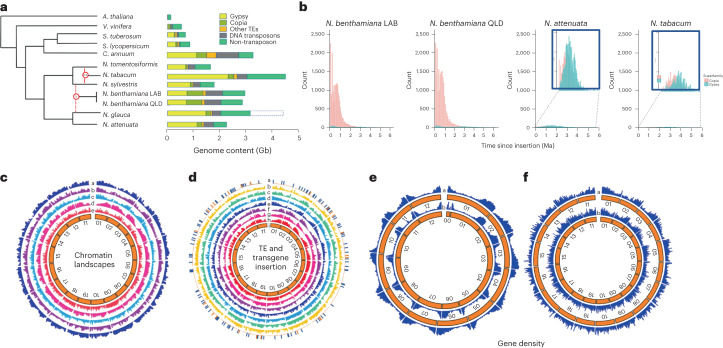
Transposon, epigenetic landscapes and gene density of *N. benthamiana*. **a**, Relative complements of transposon and non-transposon content in *Arabidopsis* *thaliana*, *Vitis vinifera* and key Solanaceous and *Nicotiana*s are presented as their calculated genome content in Gb. The dashed box for *N. glauca* indicates the genome size calculated from *k*-mer analysis (4.5 Gb), whereas the composition of the genome is based on the current assembly of 3.2–3.5 Gb. Many Gypsy-like sequences are present in the ‘other TE’ category in *N. benthamiana*. **b**, Estimated dates of LTR-retrotransposon insertion, calculated by sequence comparison between the LTRs of individual element insertions, in *N. benthamiana* LAB and QLD, compared with *N. attenuata* and *N. tabacum*. A clear and ongoing large burst of Copia element activity is evident in both LAB and QLD, which is absent in both *N. attenuata* and *N. tabacum*. The reported burst of Gypsy activity in *Nicotiana*s appears to predate the 6 Ma limit of our analysis. **c**, A Circos plot depicting the chromatin landscape compared with gene content in LAB. Tracks a and b represent respectively the location of permissive histone marks H3K27ac and H3K4me3 within each LAB chr. Track c depicts the gene density across the LAB genome, whereas tracks d and e represent the location or repressive histone marks H3K9me2 and H3K27me3, respectively. **d**, Circos plot depicting the comparative locations of transgene insertions, LTR-retrotransposon insertion and methylation marks across LAB chromosomes. Track a, transgene insertion sites; red ‘ticks’ represent insertions derived from stable transformation, blue ‘ticks’ represent insertions derived from transient agro-infiltration. Track b, insertions of intact Copia TEs (containing matching LTRs and complete internal sequences). Track c, insertion of all annotated Copia TEs, including fragmented elements. Track d, distribution of CHH methylation marks. Track e, gene density across the LAB genome. Track f, insertions of all annotated Gypsy TEs, including fragmented elements. Track g, distribution of CG methylation marks. Track h, distribution of CHG methylation marks. The innermost circle represents the numbered chromosomes. **e**, Distribution of gene densities on the chromosomes of potato (inner circle) and tomato (outer circle). **f**, Distribution of gene densities on the chromosomes of LAB (inner circle) and QLD (outer circle) genomes.

Polyploidization is often accompanied by bursts of transposable element (TE) activity^39–42^ and TEs, especially the type 1 LTR class such as Gypsy metavirus (Gypsy), are highly abundant in *Nicotiana*^34^. Although Gypsy proliferation is obvious in the *N. benthamiana* genome, its content (~1.5 Gb) is more similar in size to those of the diploid *Nicotiana* species than to the allotetraploid *N. tabacum* or the combined sum of the extant ancestral parental diploid relatives, *N. glauca* and *N. sylvestris* (Fig. 5a). A similar expansion of Gypsy content is evident in the recently reported pepper genome and is one of the main causes for its increased size^43^. However, as a percentage of genome size, all of these *Nicotiana*s, including *N. benthamiana*, are about 50% Gypsy or Gypsy-like sequence, suggesting that the decreased Gypsy content in *N. benthamiana* is due to whole chromosome loss rather than TE-mediated genome purging^44,45^.

**Extended Data Fig. 8:**
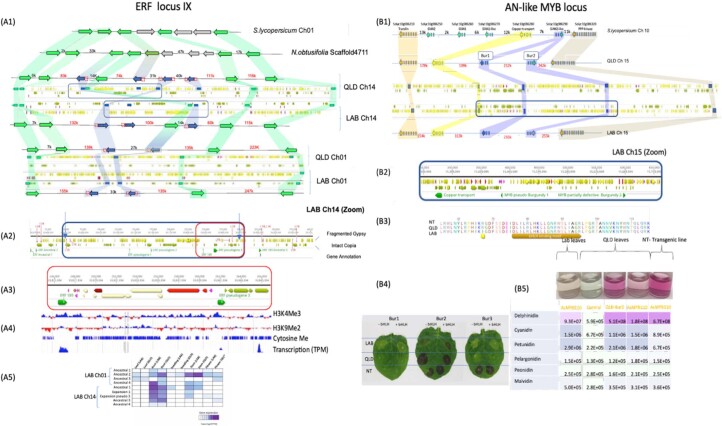
Comparison of ERF locus IX and AN-like MYB loci in LAB and QLD with other Solanaceae. **(A1)** Synteny analysis of the ERF locus IX in tomato, *N. obtusifolia*, LAB and QLD shows lineage-specific tandem duplications of ERF189s, advanced diploidization through loss of gene function, and an inversion between LAB and QLD on chr 14, flanked by newly inserted Copia elements. Functional genes are shown in green; nonfunctional/pseudogenes are in blue. Gypsy, Copia and LTRs are indicated as yellow, olive green and red arrows respectively. Shading indicates the orthology relationships of ERF189 genes between different syntenic blocks. The inverted region of LAB chromosome 14 and the Gypsy and Copia landscape within the blue box is magnified in the second panel (**A2**). The third panel (**A3**) is further magnifying the region indicated by a red box in (A2). The fourth panel (**A4**) depicts the epigenetic landscape (H3K4me3, H3K9me2 and cytosine methylation) and the expression of selected ERF189 genes in LAB. For H3K4me3, H3K9me2 enriched regions are shown in blue and the lack of histone modification is in red. Methylated cytosines are shown as blue bars. (**A5**) Tissue-specific gene expression of Ancestral (the left-most and right-most two genes indicated in green in *N. obtusifolia*) and ‘Expansion’ (the three green genes in the middle of *N. obtusifolia*) genes. **(B1)** Synteny analysis of the AN-like locus in tomato, LAB and QLD shows tandem duplication of SlAN2-like MYB genes in LAB and QLD with loss of gene function of 1 copy in QLD (Bur1) and both copies (Bur 1 & 2) in LAB. Loss of Bur2 in LAB is associated with a newly inserted Copia element. Functional genes are shown in bright green, and nonfunctional/pseudogenes are in dark green. Gypsy, Copia and LTRs are indicated as yellow, olive green and red arrows respectively. Shading indicates the orthology relationships. The Gypsy and Copia landscape within the blue box are zoomed in the second panel (**B2**) The third panel (**B3**) shows the amino acid change in LAB Bur2 which alters its bHLH binding site. (**B4**) shows the function of Bur1 is defective in LAB, QLD and NT, and that Bur2 is fully active in QLD and NT and may be partially restored by simultaneous overexpression of bHLH in LAB. Bur3 is only functional in NT. (**B5**) Levels of different anthocyanins in LAB and QLD leaves following transient expression of AcMYB110 (an AN-like MYB from Kiwifruit) or QLD Bur2. For comparison, the Anthocyanin levels were measured in NT stably transformed with an AcMYB110 construct.

**Extended Data Fig. 9:**
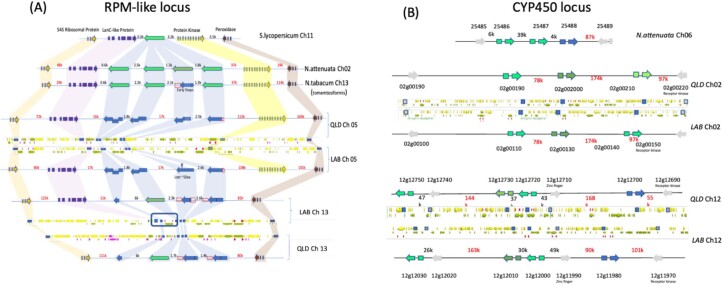
Comparison of RPM1-like locus and Cytochrome P450  loci in LAB and QLD with other Solanaceae. **(a)** Synteny of RPM1-like loci in tomato, *N. attenuata*, *N.tabacum*, LAB and QLD. **(b)** Synteny of a terpene biosynthesis pathway Cytochrome P450 locus in *N. attenuata*, LAB and QLD. Gene arrangement in cartoon form representing RPM1-like bacterial resistance genes  and CYP736A-like genes (functional - bright green), possibly functional (dark green), defective/pseudogenes (blue). In (A), distances between genes indicated (black text)< 15kbp; (red text) >15kbp and surrounding syntenic genes in are shown in orange, purple, yellow and brown. Orthology/homology relationships are indicated by coloured shading. In (B), distances between genes indicated (black text)< 50kbp; (red text) >50kbp. TE annotation tracks for LAB and QLD were prepared using annotation data from the EDTA TE annotation pipeline (see online Methods) and Geneious Prime software (Geneious Prime 2023.0.1; https://www.geneious.com). Only LTR-transposable elements are shown. Yellow blocks represent GYPSY elements and green blocks represent COPIA elements. The size of each block is proportional to the number of base-pairs annotated for that element. Red triangles represent LTR repeat regions that flank either a GYPSY or COPIA element. These elements are likely to be nearly complete and can be considered possible autonomous elements. The rectangular red blocks flank unknown LTR-TE elements. Unknown TEs are elements that are recognized as an LTR element but are not able to be classified as either a COPIA or GYPSY element due to irregularities in internal sequences for that element. These are likely to represent non-autonomous elements. Those elements not flanked by LTR sequences are highly fragmented nonfunctional elements. The blue rectangular boxes highlight the location of the genes annotated in the tracks above and below the TE annotation tracks.

Unlike any other sequenced Solanaceous species genome, including the closely related diploid *N. attenuata* and the polyploid *N. tabacum*, the *N. benthamiana* genome shows evidence of dramatic, recent Copia element proliferation (Fig. 5a,b). Examining in more detail four different loci in the subgenomes of LAB and QLD and comparing them with their counterparts in tomato and other *Nicotiana*s (Extended Data Figs. 8–10) revealed a common theme of expansion of intergenic regions in *Nicotiana*s compared with tomato, which, as in pepper, is largely because of Gypsy elements which are now highly fragmented. A second theme is tandem duplication in *Nicotiana*, followed by extensive pseudogenization specifically in *N. benthamiana*. An abundance of recent, intact Copia elements is also evident in *N. benthamiana*. Insertion dating (Fig. 5b) reveals that sustained periods of Copia mobility started around 2 Ma, reaching a peak around 750 thousand years ago (ka), and are still occurring. This coincides with the divergence of LAB and QLD, dated at ~800 ka (ref. ^14^), and recently inserted Copia elements are evident in close proximity to key genes in all four loci that we examined (Extended Data Figs. 8–10) suggesting that the recent mobility has played a major role in the genome’s advancing diploidization and diversity. It is possible that the Copia explosion is common to all of the Australasian *Nicotianas* and, in conjunction with their allopolyploidy, this has possibly fuelled the adaptation enabling the widespread success of the *Suaveolentes* across some of the harshest climatic and ecological regions in Australia.

### Epigenetic landscape and sites of transgene integration

The epigenetic landscape of the LAB genome was examined for histone H3 methylation and acetylation, and cytosine methylation (Fig. 5c,d, and Supplementary Fig. 9)^46^. Chromosomes 1, 2, 3, 4, 5, and to a lesser extent, 11 and 12, have a pronounced gradient of gene density across each chromosome, which helps to reveal the correlation of high gene density with high levels of active histone marks (H3K4me3, H3K27ac). An inverse correlation of high gene density with repressive histone and DNA marks (H3K9me2 and CG and CHG methylation) is also apparent. These epigenetically repressed regions contain high levels of fragmented Gypsy elements, whereas the active regions correlate with increased levels of intact Copia elements. The associations are also visible in the other chromosomes at a more localized level. The remarkably high level of recent Copia element insertions into regions with high gene density and active histone marks also correlates with high levels of CHH methylation which are probably driven by active transcription of these TEs.

To investigate whether epigenetic landscape has an influence on transgene insertion in the *N. benthamiana* genome, stable transgenic lines and leaf patches agro-infiltrated with transgene-encoding constructs were analysed for their insertion locations. From 40 independent transgenic lines, 23 sites could be mapped, and whole-genome sequencing of the infiltrated patches identified 144 integration sites (Fig. 5d). When adjusted for chromosome size, there was no significant bias for integration into any specific chromosome (*P* = 0.19). However, integration into the gene body and promoter elements was more frequent than random (Supplementary Fig. 10) and those inserting into intergenic regions were significantly closer to the gene borders (Supplementary Fig. 11). Transgene insertion into the gene body was at a much higher rate in transiently agro-infiltrated tissue than in stable transgenic lines, presumably because insertion-mediated dysfunctionality of some genes prevents whole-plant regeneration but is not lethal in confined patches of somatic tissue. The average intergenic size for *N. benthamiana* is ~60 kb (Supplementary Fig. 12) and the majority of transgenes have been inserted within the 10 kb region adjacent to a gene. A similar bias is apparent for active copies of both Copia and Gypsy (Fig. 5d and Supplementary Fig. 11). Coupled with the histone and cytosine methylation status data, this supports the notion that transgenes and TEs are more able to integrate into the open chromatin of genes and adjacent regions than into the condensed core of intergenic zones.

### Diploidization and pathway dysfunction in *N. benthamiana*

The loss of five chromosomes from the ancestral allotetraploid with retention of ~50% of the genes in the genome as singletons (LAB sgA: 10,075 sgB: 11,906; QLD sgA: 11,416 sgB: 12,905) rather than homeologous pairs (Fig. 2d and Supplementary Table 11,a–h), indicates a loss of ~20,000 genes/genome over 5 Myr. This complies with the estimation that the ancestral allotetraploid genome had ~70,000 genes^31,32^ and, coupled with LAB’s genetic dysfunctions, explains the simple 3:1 Mendelian inheritance ratios of many traits in LAB × QLD crosses, such as virus susceptibility^14^, nornicotine production and anthocyanin competence. In each of these, LAB has dysfunctional genes and pathways compared with QLD. The anthocyanin-regulating transcription factor (TF) locus shows tandem gene duplication with progressive gene dysfunction (Extended Data Fig. 8(B)). Even more striking diploidization is apparent in the nicotine synthesis regulating *ERF IX* TF locus (Extended Data Fig. 8(A)), the *RPM1*-like bacterial defence gene locus (Extended Data Fig. 9(A)) and the terpene biosynthesis *CYP736A* gene locus (Extended Data Fig. 9(B)). In all of these, there is evidence of recently inserted Copia elements, suggestive of their role in the process. Diploid *Solanum* genomes and many non-Solanaceous species exhibit high gene density bias towards the chromosome termini (Fig. 5e,f). Interestingly, *N. benthamiana* chromosomes, especially 5–10 and 15–19, have a more uniform density. This unusual arrangement was probably caused by their formation through abundant inter-chromosomal recombination and by gene density dilution through the favoured insertion of TEs into the active chromatin of gene-rich regions.

## Discussion

The exponential adoption of *Nicotiana benthamiana* as a model plant over the past two decades has produced vast amounts of data describing its responses to a wide spectrum of biotic and abiotic challenges, and this seems likely to continue unabated. Its use as a bioplatform to produce therapeutics has a similar trajectory. This dual role as a model species and non-food bioproduction platform, on top of the unmatched capacity for fast transient transgene analysis, has made *N. benthamiana* the chassis of choice for testing and implementing the most advanced engineering approaches in plant synthetic biology^47–49^. We have produced a high-quality genome assembly of the LAB strain of *N. benthamiana* with fully annotated gene models, miRNA families, TEs, epigenetic landscapes and chromosomal subgenomic membership, and made this publicly available on an interactive web-based genome browser. This enables decades of previously obtained data to be placed in a broader context, provides an important aid for future research and biotechnology, and facilitates the involvement of the scientific community to expand and refine the resource. The high-quality genome assembly of QLD with its additional pathways and ~3,000 genes, and the details about genomic diversity of an additional four wild and two laboratory isolates, provide resources to greatly enhance metabolic, developmental and evolutionary studies. This is relevant not only to *N. benthamiana*, but also across the Solanaceae, because it brings the genome of a *Nicotiana* species to the same chromosomal level of completeness (>95%) as tomato, eggplant, potato and pepper.

Compared with QLD, LAB is defective in many pathways including viral defence owing to a dysfunctional RNA polymerase gene (*RDR1*), but both accessions have similar levels of expression and homoeologue retention for the other RNAi pathway genes. Although QLD has a greater genetic spectrum for metabolic and biotechnological engineering than LAB and similarly high transformation and gene editing efficiencies, its slower growth rate and lower yields of transiently expressed antibodies following vacuum agro-infiltration make LAB the preferred choice as a biofactory and research tool. However, QLD and LAB are highly interfertile (Supplementary Fig. 13) making them a powerful partnership for a wide range of molecular genetic and comparative genomics approaches such as recombinant inbred and epigenetic recombinant inbred populations reminiscent of well-established model plant systems such as *Arabidopsis*, maize and rice.

*N. benthamiana* shows a recent explosion of Copia mobility and rapidly advancing diploidization. These two phenomena may or may not have a cause–effect relationship, but are apparently unique to this species, among sequenced *Nicotiana*s, making it an excellent model species to study the course of diploidization and the dynamic balance of two subgenomes undergoing this process.

## Methods

### Plant lines

*Nicotiana benthamiana* LAB, NT, SA, WA, QLD and NWA accessions have been described previously^14^. The EU-LAB isolate extensively used GFP-expressing transgenic line (16c) and produced in D. Baulcombe’s laboratory, Sainsbury Institute, UK^23,27^ and USA-LAB have been described^50^. Plants were grown in a custom soil mix (UQ23 supplemented with Osmocote slow release fertilizer) under controlled environmental conditions at a constant temperature of 25 °C with a 16-h light and 8-h dark photoperiod.

### RNA-seq

Total RNA was isolated from four tissues (leaf, flower, stem, root) and seedlings (10 days) of LAB (6 weeks) and QLD (7 weeks) at the same developmental stage using TRIzol reagent according to the manufacturer’s instructions. Libraries were constructed in triplicate for each tissue using NEBNext ultra RNA Library Prep Kit for Illumina, size selected (average 300 nucleotides), and sequenced on an Illumina HiSeq 2000/2500 system to produce 150 bp paired-end reads.

### Extraction and analysis of secondary metabolites from plant tissues

Flower, leaf, stem and roots were sampled as described for RNA-seq and two biological replicates (individual plants) of the same samples of LAB and QLD were used for the metabolic analysis. Tissues were freeze-dried and homogeneously grounded in liquid nitrogen.

The semi-polar fraction was extracted from lyophilized ground tissue (3 mg for flower and root, and 5 mg for leaf and stem tissues) with 75% methanol/0.1% v/v formic acid, spiked with 0.25 µg ml^−1^ of formononetin (Sigma-Aldrich) as an internal standard. Metabolites were extracted at room temperature by continuous agitation for 30 min in MM 400 at 20 Hz. Samples were centrifuged at 20,000*g* for 20 min, and 0.6 ml of the supernatant was transferred into filter polytetrafluoroethylene vials for liquid chromatography–mass spectrometry analysis (0.2 µm pore size). Two independent extractions and analyses were performed for each biological replicate. Liquid chromatography conditions have been described previously^51^. Five microliters of the filtered extract was injected into the liquid chromatography–heated electrospray ionization–mass spectrometry system, using a Q-exactive mass spectrometer (Thermo Fisher Scientific). The ionization was performed using the heated electrospray ionization source, with nitrogen used as a sheath and auxiliary gas, and set to 35 and 10 units, respectively. The capillary temperature was 250 °C, the spray voltage was set to 3.5 kV, the probe heater temperature was 330 °C, and the S-lens RF level was set at 50. The acquisition was performed with Fourier transform mass spectrometry with a mass range of 110–1,600 m/z both in positive and negative ion mode, with the following parameters: resolution 70,000, microscan 1, AGC target 1 × 10^6^ and maximum injection time 100 milliseconds. Dd-MS2 parameters were as follows: resolution 17,500, intensity threshold 4.0 × 10^4^, AGC target 2 × 10^4^, maximum injection time 50 milliseconds, TopN 5, stepped normalized collision energy 15, 25, 40. All the chemicals and solvents used during the entire procedure were of LC/MS grade (Chromasolv, Merck Millipore).

Metabolic diversity was evaluated by comparing the MS spectra (positive ion mode) using SIEVE software (Thermo Fisher Scientific)^51^. The LC–MS spectra were processed by comparing tissues from each ecotype; only metabolites accumulating to levels of more than twofold change and *P* < 0.05 (*t*-test) between the two ecotypes were selected. Metabolites were identified based on accurate masses in full MS together with MS2 spectra and/or authentic standards, using the KEGG (https://www.genome.jp/kegg/compound/), Metfrag (https://ipb-halle.github.io/MetFrag/projects/metfragweb/) and PubChem mass databases (ST3) (https://pubchem.ncbi.nlm.nih.gov/). Relative levels of accumulation of investigated metabolites were measured and normalized relative to distilled water and the internal standard, to correct for extraction and injection variability, as described^51^.

### Whole-plant vacuum infiltration and antibody purification

Small-scale trastuzumab expression studies were performed using 5–6-week-old *N. benthamiana* plants. *Agrobacterium tumefaciens* strain GV3101 containing plasmids with expression cassettes for trastuzumab light chain, trastuzumab heavy chain, P19 and galactosyl transferase (https://www.plantformcorp.com/) were centrifuged at 12,000*g* for 30 min then resuspended in infiltration buffer to an optical density at 600 nm of 0.2. The infiltration solution was poured into 2 l beakers, filling each beaker to the rim. The aerial portions of *N. benthamiana* plants were submerged in the infiltration solution and placed in a 15-gallon vacuum chamber (Best Value Vacs, catalogue no. BVV15G). Using a vacuum line, a vacuum was applied until the pressure on the chamber reached −25 inHg, then held for 3 min and slowly released. *N. benthamiana* plants were then removed from solution and returned to the growth chamber. Leaf tissue was harvested 7 days post infiltration and stored at −80 °C until processing.

Frozen infiltrated plant tissue was homogenized in liquid nitrogen with a mortar and pestle then combined with 3 volumes of 4 °C PBS buffer pH 7.4. The homogenate was then centrifuged at 16,000*g* for 30 min at 4 °C. The total soluble protein was then passed through a 0.45 μm filter into a clean tube. The antibody was then purified according to the manufacturer’s instructions supplied with the Protein G HP SpinTrap kit (GE Healthcare, catalogue no. 28903134) using the standard purification protocol.

### Whole-genome sequencing

High molecular weight genomic DNA from leaves or leaf nuclei of *N. benthamiana* LAB and QLD ecotypes was extracted as described^52^ and used for whole-genome sequencing (Illumina, PacBio and Oxford Nanopore; Supplementary Fig. 3). Illumina and PacBio sequencing was conducted by the Central Analytical Research Facility, Queensland University of Technology (QUT-CARF) and nanopore sequencing by the Australian Genome Research Facility, Melbourne. The quality of the assemblies was determined using Merqury software (v.1.3)^53^. LTR assembly index scores were determined using the annotation obtained from the EDTA TE annotation pipeline^54^ and using the LTR assembly index sub-package of the LTR-retriever^55^ package according to Ou et al.^24^ (https://github.com/oushujun/EDTA/wiki/Calculate-LAI-from-EDTA-GFF3-files).

### Genome assembly

The assembly pipeline is summarized in Supplementary Fig. 3. LAB and QLD contigs were assembled using CANU (v.1.81)^56^ and SparseAssembler *k*-mer 77 (v.20160205)/DBG2OLC (v.20160205)/Racon (v.1.3.2)^57–59^, respectively. Bionano optical mapping^60^ gave 44 and 37 super scaffolds for LAB and QLD, respectively, with contiguity statistic N50 values of 122 and 130 Mbp. Juicer (v.1.6)^61^ and 3D-DNA (branch 201008)^62^ were used to generate Hi-C data and pre-assembly files. HiC libraries were produced as described by Dong et al.^63^, sequenced using the Illumina platform, and the aligned fragments from Juicer were further refined using Juicebox (v.2.12)^64^ and Citrus (https://github.com/anjiyuan/Citrus) to produce chromosome-level assemblies. LR_Gapcloser^65^ (v.1.1) was used to close gaps with long reads to complete our genome assemblies. Afterwards, both assemblies were polished with Illumina reads using Pilon^66^ (v.1.23). Finally, Mercury^53^ (v.1.3) was used to categorize assembly quality based on the Earth Biogenome Project^25^. First, *k*-mer for DNA Illumina sequence was generated by running the tool with ‘meryl *k* = 21 count output xxx.meryl xxx.fastq.gz’ and then generating *k*-mer completeness and quality value with ‘merqury.sh xxx.meryl <gene fasta> <prefix-output>’. The bioinformatic analyses were performed at the High-Performance Computing (HPC) facility, QUT, and on Flashlite on QRIScloud, Australia.

### Gene annotation

HISAT2 (v.2.1.0)^67^ generated Binary Alignment Map (BAM) files using pooled RNA-seq data (leaf, root, stem and seed) and Scallop (v.0.10.5)^68^ was used to identify transcripts from the pooled RNA-seq data. Transdecoder (https://github.com/TransDecoder/TransDecoder/) identified the coding and UTR regions. AUGUSTUS (v.3.2.3)^69^ was used to predict all possible transcripts based on the genome sequence. Combining the two gene annotations^70^, gave 267,000 and 255,000 genes for LAB and QLD, respectively. To filter out low-confidence predicted genes, coding sequences of all the predicted genes were BLAST-searched^71^ against the National Center For Biotechnology Information (NCBI) NR (non-redundant) gene database and Solanaceae plants (tomato, potato, *N. attenuata*, *N. tabacum*) with the ‘identity’ parameter gradually reduced until the BUSCO (v.4.0.5)^72^ score did not increase. These were identity values of 86% (LAB) and 83% (QLD). To simplify the gene annotation, only one isoform (containing the longest CDS) was retained where there appeared to be overlapping genes. Supplementing these high-confidence genes with those lost in the analysis but identified by Scallop gave 45,796 and 49,636 genes for LAB and QLD, respectively. Gene mapping was undertaken by BLAST searching Tomato (https://solgenomics.net/ftp/tomato_genome/assembly/build_4.00/, v4.0), *N. attenuata* (https://www.ncbi.nlm.nih.gov/assembly/GCF_001879085.1/, including scaffolds) and *N. tabacum* (https://solgenomics.net/ftp/genomes/Nicotiana_tabacum/edwards_et_al_2017/) genomes with the sequences of gene coding regions from the LAB genome. Default BLAST settings were used.

### Protein cluster analysis

Orthofinder v.2.5.4 (ref. ^73^) (using default settings) identified orthologous relationships among LAB, QLD, identified *N. tabacum*, *N. sylvestris*, *N. tomentosiformis*, *N. glauca*, *A. thaliana, V. vinifera, Solanum lycopersicum and S. tuberosum*. The UpSet plot in Supplementary Fig. 9 is generated using UpSetR package^74^. See Supplementary Table 7c for details about the genomes used.

### TE annotation

The EDTA pipeline (v.2.0.0)^54^ (https://github.com/oushujun/EDTA); last accessed 22 September 2022) was used to annotate the repeat element space for LAB, QLD, *N. attenuata* and *N. tabacum* with the following initiating command:

>EDTA.pl-genome <genome fasta>-species others -step all -u -sensitive 0 -anno 1 -threads 48.

The annotation of the *N. tabacum* genome only made use of the chromosome assembly available from the Sol Genomics Network (https://solgenomics.net/organism/Nicotiana_tabacum/genome; file Nitab-v4.5_genome_Chr_Edwards2017.fasta.gz). The -u flag generates a file (*EDTA_raw/LTR/*.pass.list), containing estimations of LTR insertion times from LTR-retriever^55^ a component part of the EDTA pipeline. The estimation of insertion time is based on the number of polymorphisms calculated between the LTR sequences of intact long terminal repeat transposable elements. Because of the lack of an accurate estimation of the neutral mutation rate in *N. benthamiana*, the default rate was set to that calculated for rice: 1.3 × 10^−8^ substitutions per base pair per year^54^.

### MicroRNA annotation

The mature miRNA sequences from 79 plant species (Supplementary Table 8e) were retrieved from miRbase (release 21; https://www.mirbase.org/) and used to identify microRNAs (miRs) in *N. benthamiana* using bowtie (v.2.0)^75^. To avoid missing IsomiRs, possible mature miRNA sequences with one mismatch were also identified using miRPlant (v.6)^76^. The expression levels of each miR and its precursor transcript were calculated from pooled data of libraries of small RNA and RNA-seq reads (from this and previous studies^77,78^).

### SNP calling

All Illumina genomic paired-end reads from each ecotype were aligned to the LAB and QLD assemblies using bowtie2 (v.2.3.5)^79^. Duplicate reads were removed from each BAM file with Picard toolkit (https://broadinstitute.github.io/picard/) (v.2.19), MarkDuplicates (picard -Xmx25g MarkDuplicates ASSUME_SORT_ORDER=coordinate REMOVE_DUPLICATES=true), and SAMtools (v.1.10)^80^ was used to keep unique (samtools view -Sb -q 40) and proper pair-end reads (samtools view -@ 1 -hb -f 0 × *2 -F 2316*). Each read ID in the BAM file was modified by adding the ecotype’s ID using generate_subset_BAM.py from the SGSautoSNP^28^ pipeline (v.2.001). Next, BAM files for each cultivar were merged using SAMtools to produce BAM files for LAB and QLD. Finally, The SGSautoSNP.py script was used with default parameters.

### Chromatin immunoprecipitation sequencing

Cross-linking, chromatin isolation, nuclei lysis, chromatin shearing and immunoprecipitation were carried out as described by Ranawaka et al.^52^. Antibodies against two active histone marks, anti-histone-H3-tri-methyl-K4 (Abcam, catalogue no. ab8580) and anti-histone-H3-acetyl-K27 (Abcam, catalogue no. ab4729), and two repressive histone marks, anti-histone-H3-tri-methyl-K27 (Abcam, catalogue no. ab6002) and anti-histone-H3-di-methyl-K9 (Diagenode, catalogue no. C15410060) were used in the immunoprecipitation step to generate the genome-wide histone modification landscapes of LAB and QLD. Libraries (two replicates per histone modification and control input) were prepared using NEBNext Ultra II DNA Library Prep Kit for Illumina (catalogue no. E7645S) as per the manufacturer’s specifications. Chromatin immunoprecipitation sequencing libraries of H3K9me2 were sequenced at QUT-CARF, using Illumina NextSeq 500 with the output of 75 bp paired-end reads (TG NextSeq 500/550 High Output Kit v2, 75 cycle, TG-160-2005). Libraries of H3K4me3, H3K27me3 and H3K27ac were sequenced at Novogene International Private Limited (Singapore) on the Illumina HiSeq 2000/2500 system to produce 150 bp paired-end reads and analysed using the Galaxy platform (https://usegalaxy.org.au)^81^. Paired-end reads were aligned against LAB and QLD genome assemblies using bowtie2 (v.2.4.2) with default settings^75^. Alignments with mapping quality of < 40 were discarded before downstream analyses to ensure homeologue specificity and accuracy. The deepTools, bamCompare^82^, was used to quantify and visualize histone marks across genes.

### Whole-genome bisulfite sequencing

Whole-genome bisulfite sequencing samples were prepared with genomic DNA extracted from the same tissues used for chromatin immunoprecipitation sequencing. Leaf genomic DNA from three replicates was extracted using a DNeasy Plant Mini Kit (QIAGEN, 69104). The bisulfite conversion of the DNA was carried out using the EZ DNA Methylation-Gold kit (ZYMO, D5005), and the bisulfite-treated DNA libraries were constructed using the Illumina TruSeq DNA sample prep kit, following the manufacturer’s instructions. The library preparation and the subsequent next-generation sequencing were completed by Novogene HK Company Limited (Hong Kong Subsidiary). Paired-end read (150 bp) sequencing of the bisulfite-treated DNA libraries was performed using an Illumina HiSeqX system.

### Methylation analysis

The high-quality reads from whole-genome bisulfite sequencing samples were aligned to LAB and QLD genome assemblies using the default settings of the Bismark program (v.0.19.0)^83^. PCR duplicates were removed with the deduplicate_bismark implemented in the Bismark program (v.0.19.0). Reads were mapped to the non-methylated chloroplast genome as a control to calculate the sodium bisulfite conversion rate of unmethylated cytosines which was >99.9% for all replicates (three replicates from each LAB and QLD). The cytosine methylation level was calculated using the bismark_methylation_extractor in Bismark (v.0.19.0). The methylation ratio of cytosine was calculated as the number of methylated cytosines divided by the number of reads covering that position.

### Calculation of relative expression levels of A and B subgenome homeologues

The MCScanX toolkit^84^ was used to identify intraspecies syntenic blocks using protein sequences and chromosomal locations of genes (e value 1 × 10^−10^, max-target-seqs 6, masking 1, max-hsps 1). SynVisio^85^, an interactive multiscale synteny visualization tool for McScanX, was used to visualize the gene-level collinearity. Genes in syntenic blocks were identified as homeologues, and the genes that could not find their homoeologous partners were identified as singletons. The average transcripts per million (TPM) expression of genes in each tissue type was calculated (average expression per tissue). Then, using the average expression of each gene per tissue, the global expression across all tissues was calculated. Global expression >0.5 TPM was used for downstream analysis. Values of this combined analysis were used to determine the relative expression of homeologues. The homoeologous pairs were defined as expressed when the sum of the a and b subgenome homeologues was >0.5 TPM. This filtration included duplicate pairs in which only a single homeologue was expressed. To standardize the relative expression of homeologues, the absolute TPM for each gene within the duplicate pair was normalized as follows. A and B represent the genes corresponding to the A and B homeologues in pairs.

Relative expression of A = TPM(A)/(TPM(A) + TPM(B))

Relative expression of B = TPM(B)/(TPM(A) + TPM(B))

The Kruskal–Wallis test was performed to statistically determine the homoeologue expression bias between subgenomes. Overrepresentation analysis was conducted using Fisher’s exact test. All the genes in *N. benthamiana* were BLASTed, mapped and annotated using the Blast2Go suite^86^ and used as the background for the overrepresentation analysis. Highly suppressed genes in both subgenomes were assessed. Genes with a *P* value <0.05 were considered significantly overrepresented.

### Identification and phylogenic analysis of *ERF189*, *NBS-LRR RPM1*-like, anthocyanin *R2R3 Myb* and nicotine demethylase *CYP82RE* genes

*ERF189*, *NBS-LRR RPM1*-like, anthocyanin *R2R3 Myb* and *CYP82* genes in *N. benthamiana* were identified based on sequence homology using *N. attenuata* protein sequences (http://nadh.ice.mpg.de/NaDH/others/data) as query sequences for the tBLASTn function on Apollo (https://www.nbenth.com). *N. attenuata* CYP82 (NiAv7g20333) was identified by sequence similarity to tobacco *CYP82E4*, a demonstrated nicotine demethylase gene^87^. Phylogenetic trees were built using the identified nucleotide sequences and their available counterparts in other *Nicotiana* species (*N. attenuata*, *N. tabacum*, *N. sylvestris*, *N. tomentosiformis*) aligned using Muscle (v.3.8)^88^. The best nucleotide substitution model was estimated based on jModeltest2 (v.2.1)^89^ and a tree constructed for each gene family using MrBayes (v.3.2.6)^90^.

### Transgene insertion analysis

*Agrobacterium tumefaciens* (GV3101) transformed with a 35s-GFP-OCS construct (pBEN0317) was infiltrated into 4-week-old *N. benthamiana* leaves. After 5 days, agro-infiltrated leaves were collected. Total genomic DNA was extracted using the ISOLATE II Plant DNA Kit Bioline (BIO-52070) and pooled before library preparation using TruSeq DNA Library Prep Kits (FC-121-2001). Sequencing was performed using the Illumina HiSeq 2000 platform. Paired-end reads were mapped to pBEN0317 binary vector using Burrows–Wheeler Aligner (BWA-MEM) (v.0.7)^91^. To determine the transfer DNA integration events, all split reads that partially overlapped the T-DNA region’s left and right borders were extracted and searched using BLASTn against the *N. benthamiana* genome. Reads with an identity higher than 85% and an e value less than 1 × 10^−5^ were selected as high-confidence transgene integration sites. A different approach was used to identify the broken reads. Reads were initially mapped to the *N. benthamiana* genome and mapped reads whose mate is unmapped were extracted using Samtools view^80^. The filtered BAM file was converted to fastq using bedtools Convert BAM to FastQ^92^. Reads were then BLASTed to the pBEN0317 vector. The reads which mapped to vectors with an e value of less than 1 × 10^−5^ and more than a 100 bp alignment were then BLASTed to the *N. benthamiana* genome. Reads with high identity (>95%) and >50% coverage were identified as integrated T-DNA in the plant genome. For the stable transformation analysis, leaf tissues were collected from 5-week-old *N. benthamiana* stable transgenic independent lines generated using pFN117 (Cas9) and pUQC-GFP-(218). Genomic DNA was extracted following the cetyltrimethylammonium bromide method. Nested, insertion-specific primers for the right borders (RB1, RB2 and RB3 RB2 and RB3; Table 2) of pFN117 and pUQC-GFP-(218)-A were designed. Arbitrary degenerate primers and the high-throughput thermal asymmetric interlaced polymerase chain reaction (ht-TAIL-PCR) program were as described by Singer and Burke^93^. Purified PCR products were directly Sanger sequenced using RB3 primer, and the insertion sites were identified through a BLASTn search against the *N. benthamiana* genome. The number of stable and transient T-DNA insertion sites that intersect gene body, promoter, terminator and TEs were determined using the bedtools Intersect tool (v.2.30.0)^92^ and the length to the closest gene from the insertion site was calculated using RnaChipIntegrator (v.1.1.0) (https://github.com/fls-bioinformatics-core/RnaChipIntegrator). The *z*-score test for two population proportions was used to determine the significant difference between 10 kb, 10–20 kb, 20–30 kb and 30–40 kb intervals from all stable, transient transgene insertion sites and randomly selected sites in the *N. benthamiana* genome.

**Table 2:** Primers and guide RNA sequences used

Locus	Forward 5′–3′	Reverse 5′–3′
**Primers**		
NbCYP82E2	TCCACTTCAATAACGACGGC	CGCCGTAAAGAAAAGCTGGA
LABCYP82E2 promoter	TTTAAATGGCCATATCAGAGATG	TTATGAATTTTTGGATAAGAATC
QLDCYP82E2 promoter	AAACCGCGGTTAAATGGCCATATCGGAG	AAACTCGAGTATGAATTTTTGGATAAGAATC
NbGAPDH qPCR internal standard	CACTACCAACTGCCTTGCAC	ATGAAGCAGCTCTTCCACCT
pUQC-GFP-(218)-A Right Border 1	AACGCGCAATAATGGTTTCT	
pUQC-GFP-(218)-A Right Border 2	CCAAACGTAAAACGGCTTGT	
pUQC-GFP-(218)-A Right Border 3	CGCTCATGATCAGATTGTCG	
pFN117 Right Border 1	AATCCAGATCCCCCGAATTA	
pFN117 Right Border 2	CTGGCGTAATAGCGAAGAGG	
pFN117 Right Border 3	CGAATGCTAGAGCAGCTTGA	
Arbitrary degenarate primers for TAIL PCR (AD1)	NGTCGASWGANAWGAA	
Arbitrary degenarate primers for TAIL PCR (AD2)	TGWGNAGSANCASAGA	
Arbitrary degenarate primers for TAIL PCR (AD3)	AGWGNAGWANCAWAGG	
Arbitrary degenarate primers for TAIL PCR (AD6)	WGTGNAGWANCANAGA	
**gRNA sequence**		
NbRDR1	TAAATAGTACAGTTTCTCCA	
	GACACTCAAAGTTTCTCTGG	
NbRDR2	CCACTCCCAACGTAGATAAG	
	GTGTCTCGAAATGTGCTGCA	
NbRDR6	CTTACTTAGAAGTCATCAGG	
	CTGCAACAGTATTACCAAAG	
NbPDS	TCACAAACCGATATTGCTGG	
	GAGCTTCAGGAAAATCAAAG	

### Reporting summary

Further information on research design is available in the Nature Portfolio Reporting Summary linked to this article.

### Supplementary information

Supplementary Information Supplementary Figs. 1–13.

Reporting Summary

Supplementary Tables Supplementary Table 1 Morphological, developmental, and metabolic differences between LAB and QLD. Supplementary Table 2 Identification of differentially expressed semi-polar metabolites in LAB vs QLD. Supplementary Table 3 Size and number of genes in each chromosome in the LAB and QLD assemblies. Supplementary Table 4 All expressed genes in LAB. Supplementary Table 5 All expressed genes in QLD. Supplementary Table 6 Quality statistics of the LAB and QLD assemblies. Supplementary Table 7A Mapping genes in specific solanacae species with LAB to find percentage of genes in LAB with orthologues in these species. Supplementary Table 7B Protein cluster analysis. Supplementary Table 7C Characteristics and sources of genomes used for comparative analyses. Supplementary Table 8A Expressed miRNAs in LAB. Supplementary Table 8B Expressed miRNAs in QLD. Supplementary Table 8C Full list of potential miRNAs in LAB. Supplementary Table 8D Full list of potential miRNAs in LAB. Supplementary Table 8E Plant species used in miRPlant analyses. Supplementary Table 9 SNPs between different *N. benthamiana* sequenced accessions. Supplementary Table 10 Number of homeologous genes among chromosomes of LAB, *N. attenuata* and the *N. sylvestris* and *N. tomentosiformis* subgenomes of *N. tabacum*. Supplementary Table 11A Distribution of LAB homeologous genes across chromosomes of the subgenomes. Supplementary Table 11B Distribution of QLD homeologous genes across chromosomes of the subgenomes. Supplementary Table 11C Homoeologues present on partner subgenomes in LAB. Supplementary Table 11D Homoeologues present on partner subgenome but not partner chromosomes in LAB. Supplementary Table 11E Homoeologues present on same subgenome in LAB. Supplementary Table 11F Homoeologues present on partner subgenome in QLD. Supplementary Table 11G Homoeologues present on partner subgenome but not partner chromosomes in QLD. Supplementary Table 11H Homoeologues present on same subgenome in QLD. Supplementary Table 12 Homeologue expression pattern detection for subgenome dominance evaluation.

### Source data

Source Data Fig. 4 Unprocessed SDS–PAGE.

## Data Availability

The *Nicotiana benthamiana* genome and transcriptome assemblies, along with their annotations, can be accessed at https://www.nbenth.com. The raw data utilized for genome assembly and raw ChIP-seq data for the genome sequence of *N. benthamiana* LAB have been deposited in the NCBI Sequence Read Archive (SRA) under BioProject PRJNA881799. Specifically, the PacBio data for LAB and QLD can be found under the accessions SRR21820240 and SRR21820239, respectively. The HiC data for LAB and QLD are available under the accessions SRR21820238 and SRR21820237, respectively. The ChIP-seq data include SRR27031034 (K27ac), SRR27031032 (K4me3), SRR27031033 (K27me3), and their input control SRR27031035; and SRR27031030 (K9me2) and its input control SRR27031031. Databases used: KEGG (https://www.genome.jp/kegg/compound/), Metfrag (https://ipb-halle.github.io/MetFrag/projects/metfragweb/), PubChem mass databases (ST3) (https://pubchem.ncbi.nlm.nih.gov/), miRbase (release 21; https://www.mirbase.org/) and Nicotiana attenuata Data Hub (http://nadh.ice.mpg.de/NaDH/others/data). Source data are provided with this paper.

## Code availability

The code employed for obtaining chromosome-level genome sequences can be obtained from the following GitHub repository: https://github.com/anjiyuan/Citrus. The Circos plotter can be accessed via https://bioweb01.qut.edu.au/circos-bigwig/. Additionally, the synteny and dot plotter can be accessed through https://bioweb01.qut.edu.au/syntenyViewer/.
