# Author Correction: A multi-omic *Nicotiana benthamiana* resource for fundamental research and biotechnology

**DOI:** 10.1038/s41477-024-01618-x

**Published:** 2024-01-05

**Authors:** Buddhini Ranawaka, Jiyuan An, Michał T. Lorenc, Hyungtaek Jung, Maria Sulli, Giuseppe Aprea, Sally Roden, Victor Llaca, Satomi Hayashi, Leila Asadyar, Zacharie LeBlanc, Zuba Ahmed, Fatima Naim, Samanta Bolzan de Campos, Tal Cooper, Felipe F. de Felippes, Pengfei Dong, Silin Zhong, Victor Garcia-Carpintero, Diego Orzaez, Kevin J. Dudley, Aureliano Bombarely, Julia Bally, Christopher Winefield, Giovanni Giuliano, Peter M. Waterhouse

**Affiliations:** 1 Centre for Agriculture and the Bioeconomy, Queensland University of Technology (QUT), Brisbane, Queensland Australia; 2 ARC Centre of Excellence for Plant Success in Nature & Agriculture, Brisbane, Queensland Australia; 3 Italian National Agency for New Technologies, Energy and Sustainable Economic Development (ENEA), Casaccia Research Centre, Rome, Italy; 4 Genomics Technologies, Corteva Agriscience, Johnston, IA USA; 5 State Key Laboratory of Agrobiotechnology, School of Life Sciences, The Chinese University of Hong Kong, Hong Kong, China; 6 Instituto de Biología Molecular y Celular de Plantas (IBMCP), Consejo Superior de Investigaciones Científicas (CSIC), Universidad Politècnica de Valencia, Valencia, Spain; 7 School of Biology and Environmental Science, Queensland University of Technology (QUT), Brisbane, Queensland Australia; 8 QUT Central Analytical Research Facility, Queensland University of Technology (QUT), Brisbane, Queensland Australia; 9 Università degli Studi di Milano, Milan, Italy; 10 Department of Wine Food and Molecular Biosciences, Lincoln University, Lincoln, New Zealand; 11 Present Address: Centre for Animal Science, Queensland Alliance for Agriculture and Food Innovation (QAAFI), The University of Queensland, Brisbane, Queensland Australia; 12 Present Address: Centre for Crop and Disease Management, School of Molecular and Life Sciences, Curtin University, Bentley, Western Australia Australia

**Keywords:** Epigenomics, Genome evolution, Comparative genomics, Mobile elements

Correction to: *Nature Plants* https://www.nature.com/articles/s41477-023-01489-8, published online 10 August 2023.

In the version of this article initially published, the Data availability section did not list raw ChIP-seq data for the genome sequence of *N. benthamiana*. They are now included in the HTML and PDF versions of the article.

