# Supplementary material for: A multi-omic Nicotiana benthamiana resource for fundamental research and biotechnology
Source: Nat Plants. 2023 Aug 10;9(9):1558–71. doi: 10.1038/s41477-023-01489-8 (PMC10505560; doi:10.1038/s41477-023-01489-8)
Supplement: Supplementary file 1 — Supplementary Figs. 1–13. [file 41477_2023_1489_MOESM1_ESM.pdf]

nature plants

Resource

https://doi.org/10.1038/s41477-023-01489-8

A multi-omic *Nicotiana benthamiana* resource for fundamental research and biotechnology

In the format provided by the authors and unedited

Supplementary Figures

Index

Figure S1. Schematic of anthocyanin biosynthesis pathway showing genes upregulated in *N. benthamiana* after agroinfiltration with an AN-like MYB construct.

Figure S2. Schematic of assembly and annotation pipelines of the *N. benthamiana* genomes.

Figure S3. Upset plot showing orthologous groups among LAB, QLD, *N. tabacum*, *N. sylvestris*, *N. tomentosiformis*, *N. glauca*, *A. thaliana*, *V. vinifera*, *S. lycopersicum* and *S. tuberosum*.

Figure S4. Histogram showing completeness and quality of the LAB and QLD annotations based on predicted protein lengths relative to orthologs in Arabidopsis.

Figure S5. SynVisio waterfall plots showing the syntenic relationships between chromosomes of the LAB subgenomes and those of the *N. sylvestris* derived subgenome of tobacco.

Figure S6. Average relative homeolog expression in subgenomes of *N. benthamiana*.

Figure S7. RNAi associated genes

Figure S8. RDR knockout figures

Figure S9. Copia element density and methylation profiles of chromosomal regions in the proximity of genes in LAB.

Figure S10. Association of genomic features with T-DNA genomic junctions.

Figure S11. Distances to the closest gene for insertion sites for transgenes and intact Copia and Gypsy insertion sites.

Figure S12. Box and whisker plot of average intergenic distances of LAB, QLD, tomato and *N. attenuata* genomes.

Figure S13. Inter-fertility of LAB and QLD.

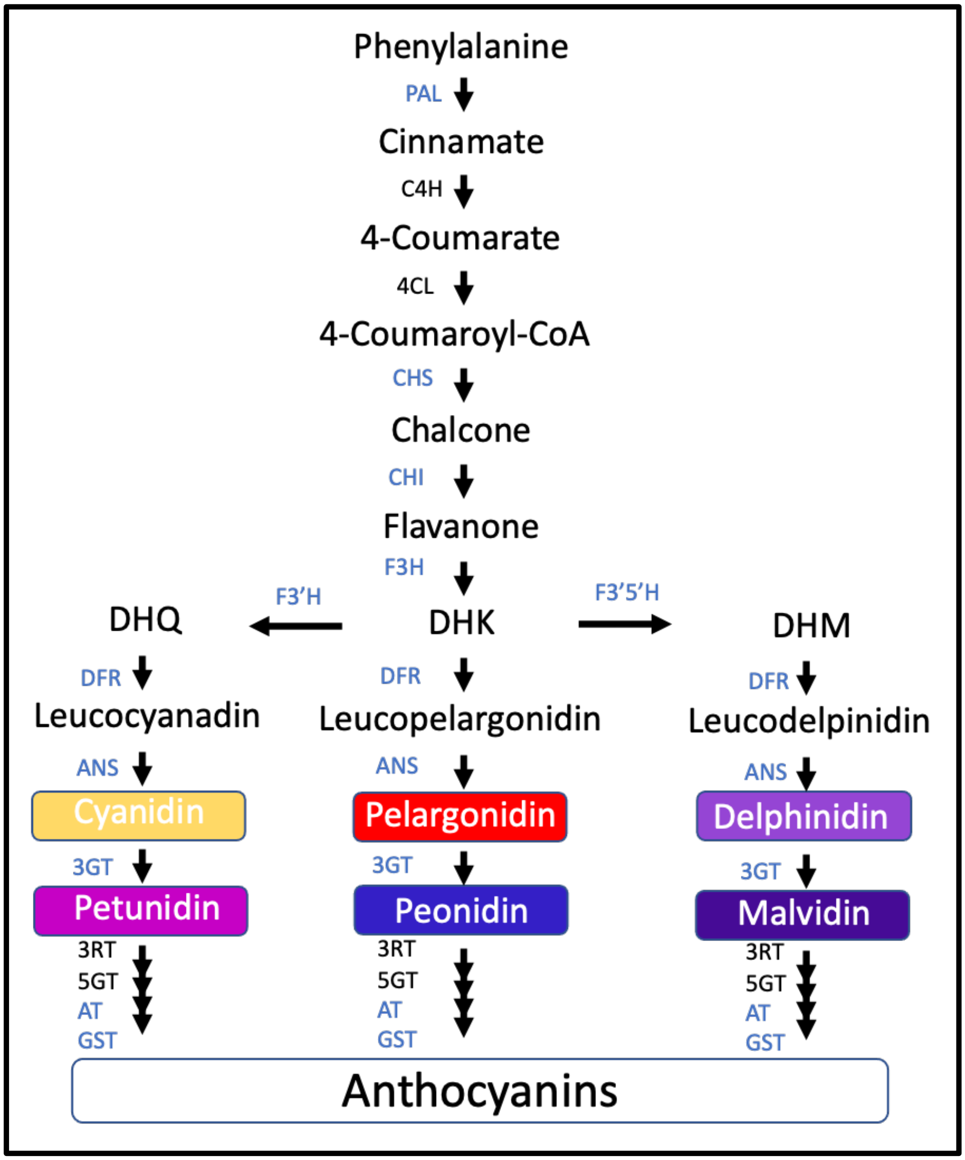

Fig. S1. The anthocyanin biosynthesis pathway with genes upregulated (shown in blue) in *N. benthamiana* five days after agroinfiltration with a construct expressing an AN-like MYB from kiwifruit.

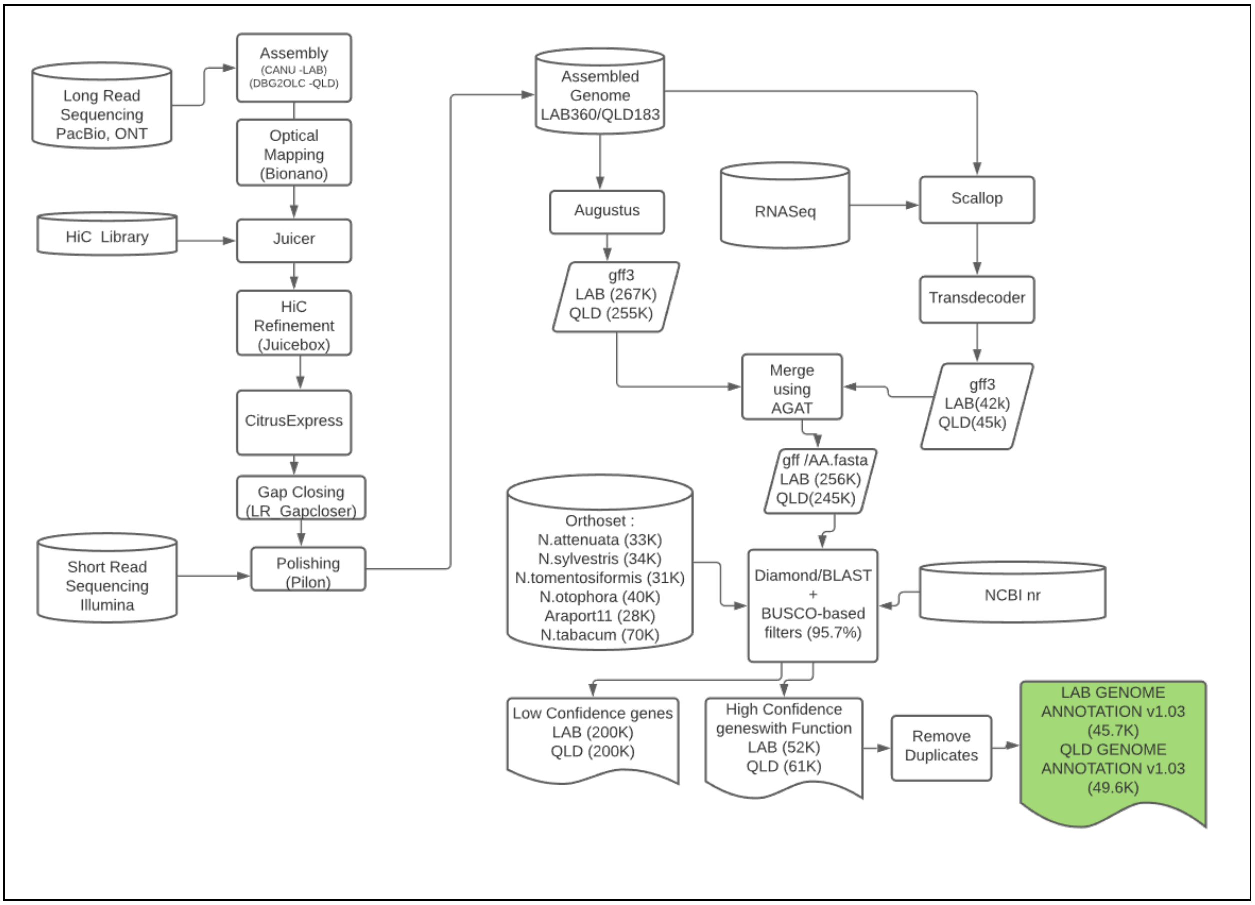

Figure S2. Assembly and annotation pipeline of the *N. benthamiana* genome based on PacBio, ONT long reads, Bionano, and chromosome conformation capture (Hi-C). DNA was extracted from purified leaf nuclei (see online methods) for both LAB and QLD. Illumina sequencing was performed on a HiSeq2500, giving ~200 Gb of paired-end 150bp reads. Long-read sequencing was performed using both PacBio (Sequel and RSII platforms; ~160 Gb per genome) and ~20 Gb ONT (Oxford nanopore R9.4). For wild accessions, 50x coverage (~150Gb) was obtained using the Hiseq2500 platform.

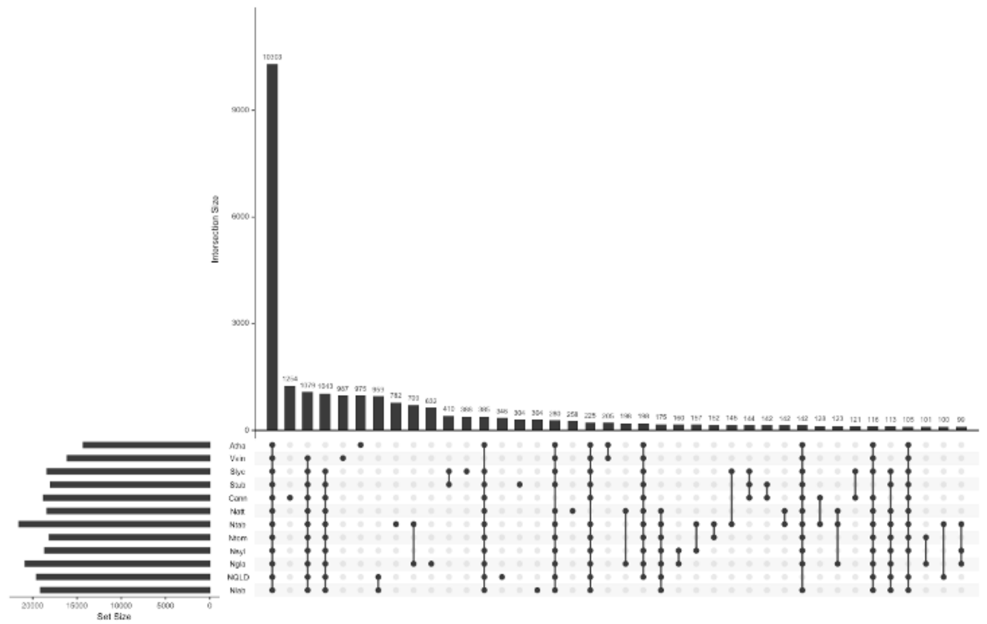

Figure S3. Upset plot showing orthologous groups among LAB, QLD, *N. tabacum*, *N. sylvestris*, *N. tomentosiformis*, *N. glauca*, *A. thaliana*, *V. vinifera*, *S. lycopersicum* and *S. tuberosum*. Filled dots (black) denote the presence, and empty dots (grey) indicate the absence of orthologous groups in each species.

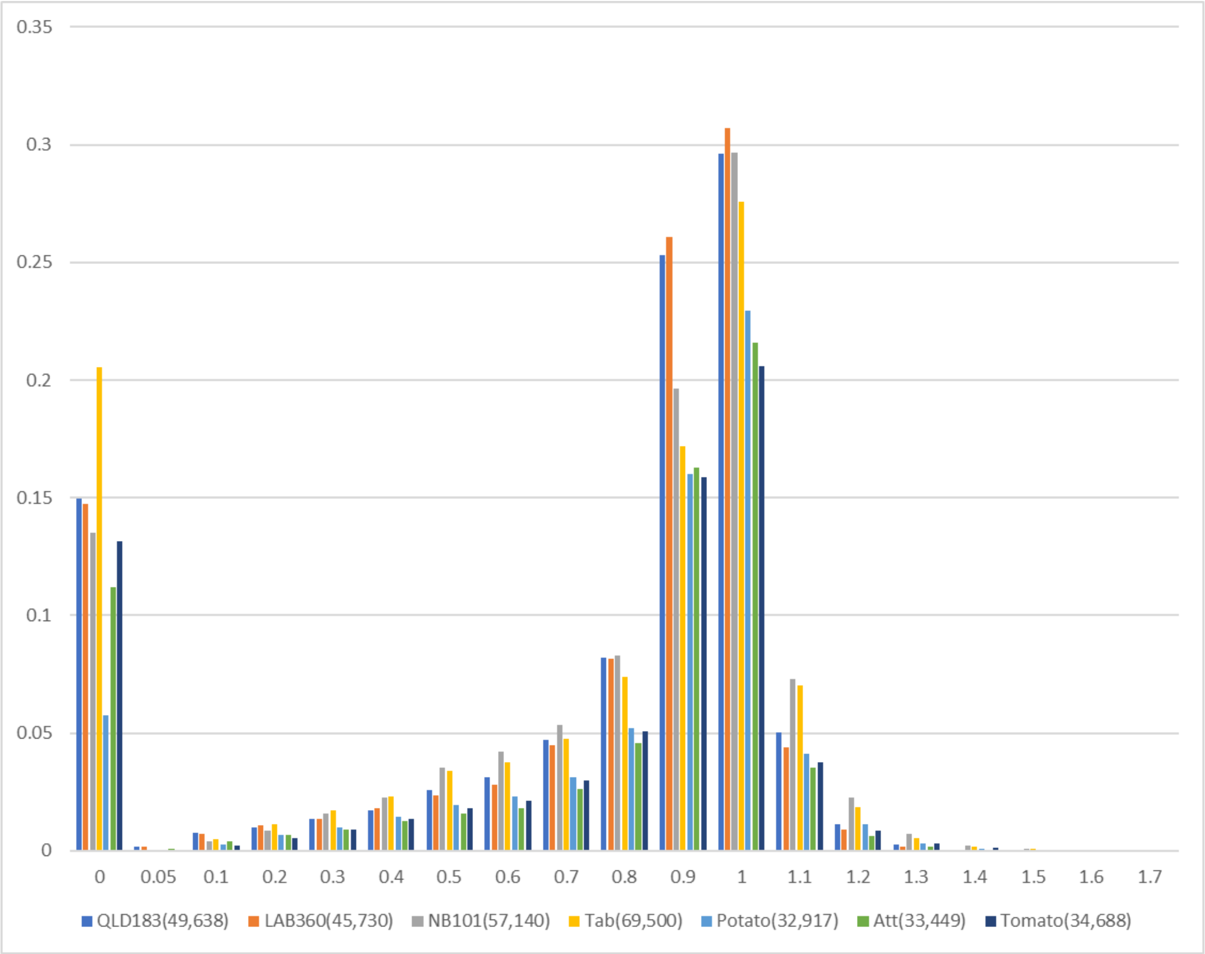

Figure S4. Completeness and quality of the LAB and QLD annotations. The predicted protein lengths of QLD, LAB, NB101, Tobacco, Potato, *N. attenuata* and Tomato were expressed as ratios compared to their Arabidopsis best hits (E-10). The number of genes in each annotation are shown in brackets. The #0 bin contains proteins that do not have an *Arabidopsis* match.

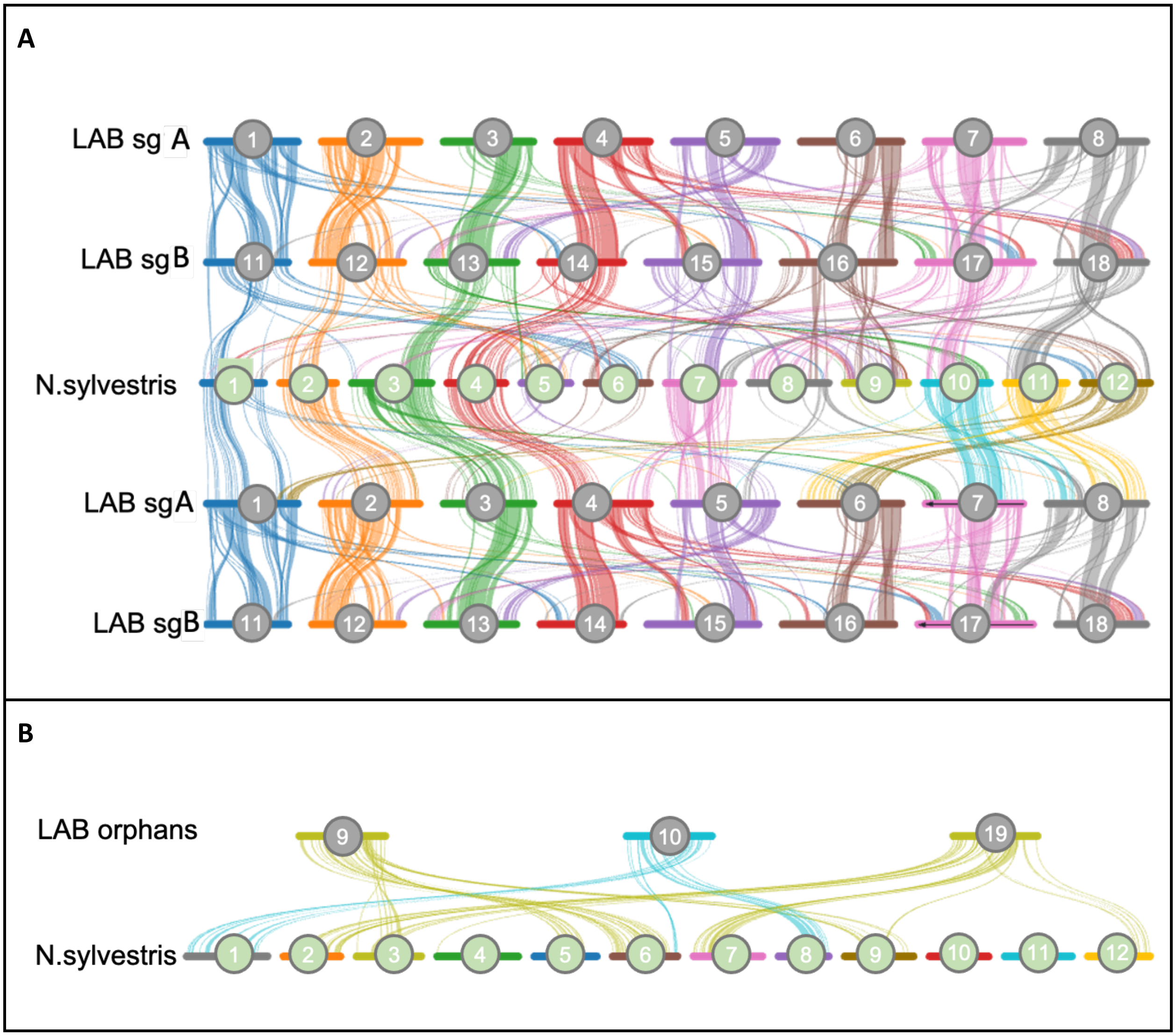

Figure S5. Waterfall plots obtained with SynVisio (https://synvisio.github.io/), showing the syntenic relationships between chromosomes of the LAB subgenomes and those of the *N. sylvestris* derived subgenome of tobacco. A). homeologous chromosome pairs. B). orphan chromosomes. The subgenomes of *N. benthamiana* have more and larger blocks of synteny with each other than with the chromosomes of *N. sylvestris.*

Relative Expression

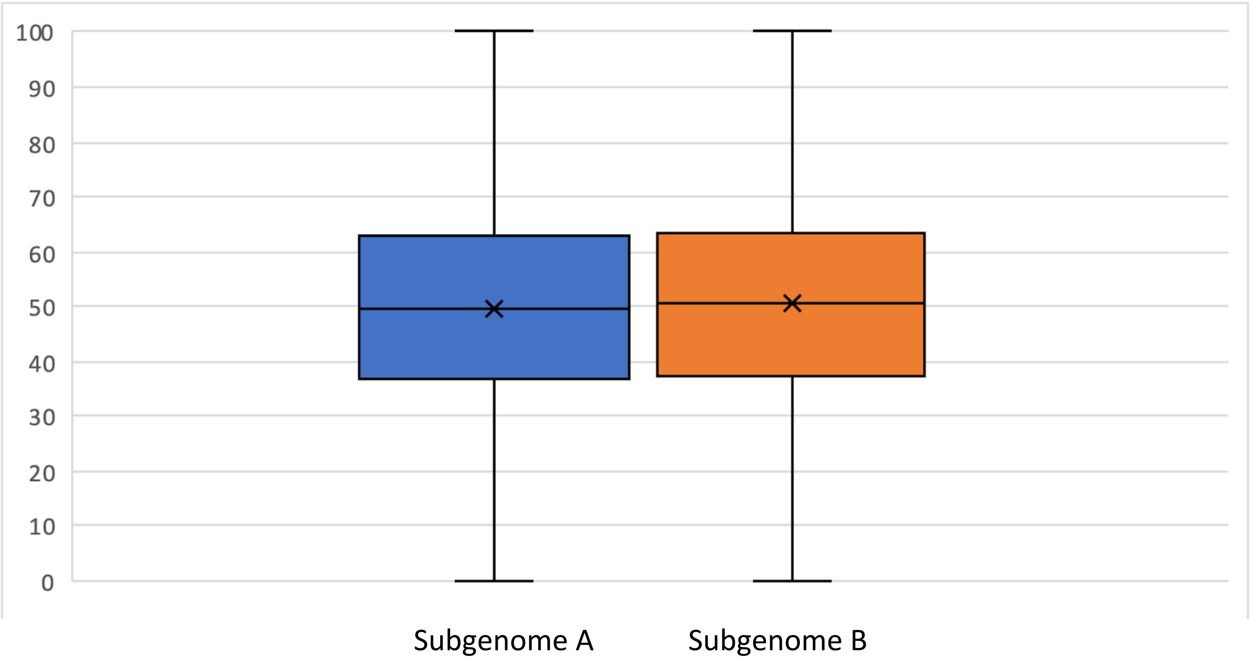

Figure S6. Average relative homeolog expression in the subgenomes of *N. benthamiana*. No significant subgenome dominance based on the homeolog expression bias was observed between subgenomes A and B (Kruskal-Wallis p value > 0.1). The analysis focused on homeologs which had a 1:1 correspondence across the two homoeologous subgenomes. To standardise the relative expression of homeologs, the absolute TPM for each gene within the duplicate pair was normalised as explained in Online methods. The relative expression was calculated using n=3 biologically independent leaf samples. Mean values are shown in each box as a cross ("X"); the line across the box indicates the median of the relative expression. Each box represents the range of relative expressions that fall into the second and third quartiles. The whiskers mark the 5th and 95th percentiles.

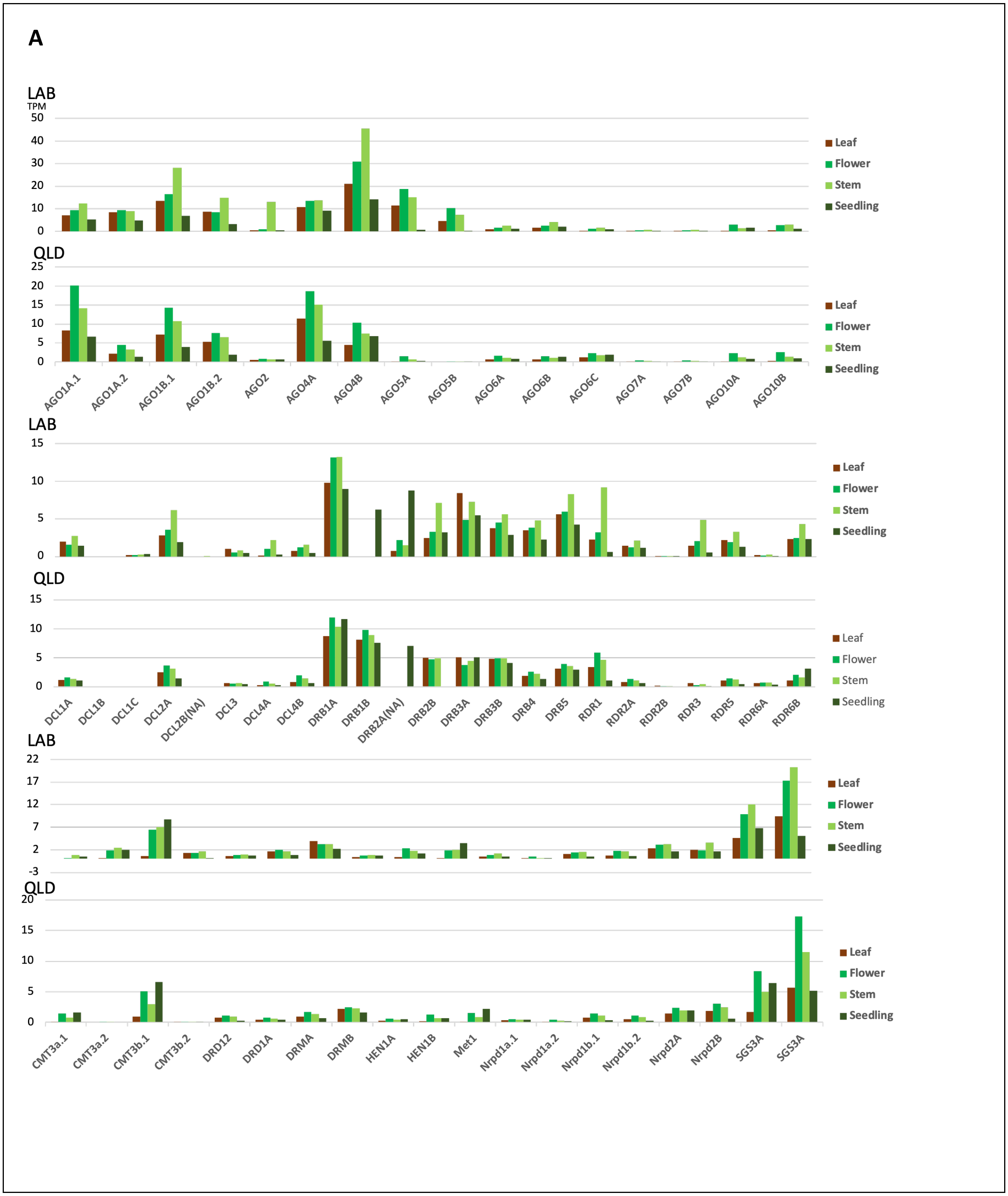
A
LAB
TPM
50
40
30
20
10
0
Leaf
Flower
Stem
Seedling
QLD
25
20
15
10
5
0
Leaf
Flower
Stem
Seedling
AGO1A.1
AGO1A.2
AGO1B.1
AGO1B.2
AGO2
AGO4A
AGO4B
AGO5A
AGO5B
AGO6A
AGO6B
AGO6C
AGO7A
AGO7B
AGO10A
AGO10B
LAB
15
10
5
0
Leaf
Flower
Stem
Seedling
QLD
15
10
5
0
Leaf
Flower
Stem
Seedling
DCL1A
DCL1B
DCL1C
DCL2A
DCL2B(NA)
DCL3
DCL4A
DCL4B
DRB1A
DRB1B
DRB2A(NA)
DRB2B
DRB3A
DRB3B
DRB4
DRB5
RDR1
RDR2A
RDR2B
RDR3
RDR5
RDR6A
RDR6B
LAB
22
17
12
7
2
-3
Leaf
Flower
Stem
Seedling
QLD
20
15
10
5
0
Leaf
Flower
Stem
Seedling
CMT3a.1
CMT3a.2
CMT3b.1
CMT3b.2
DRD12
DRD1A
DRMA
DRMB
HEN1A
HEN1B
Met1
Nrpd1a.1
Nrpd1a.2
Nrpd1b.1
Nrpd1b.2
Nrpd2A
Nrpd2B
SGS3A
SGS3A

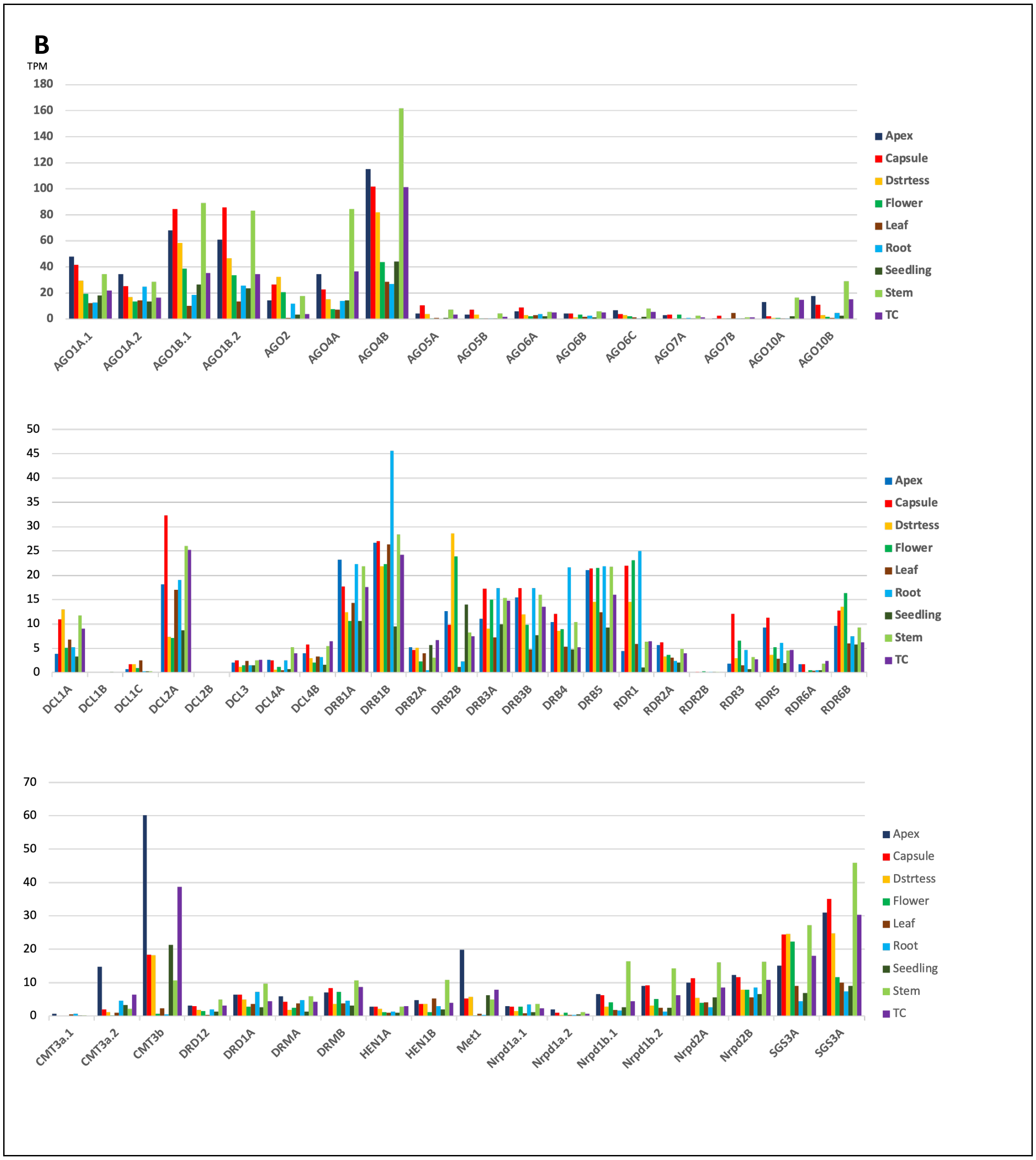

Figure S7. Expression of RNAi-associated genes in terms of TPM (Transcripts Per Million).

(A) Comparison of averaged expression (three replicates for each tissue type) of RNAi-associated genes in LAB and QLD. The first and second panels show the expression of Argonaut genes (AGO) and Dicer (DCL), Double-stranded RNA-binding proteins (DRB), and RNA-dependent RNA polymerase (RDR) respectively. The third panel shows the expression of other important genes involved in RNAi pathway. 'A' and 'B' at the end of the gene name stands for the corresponding subgenome of that gene. TPMs were calculated using the RNAseq data generated in the current study. Both LAB and QLD RNAi-associated genes follow a similar expression pattern observed in FigS7B.

(B) The figure shows the relative abundances of RNAi-associated (LAB). RNAseq data from Nakasugi et al 2013 were used in this analysis. Gene expression was examined in nine different tissues.

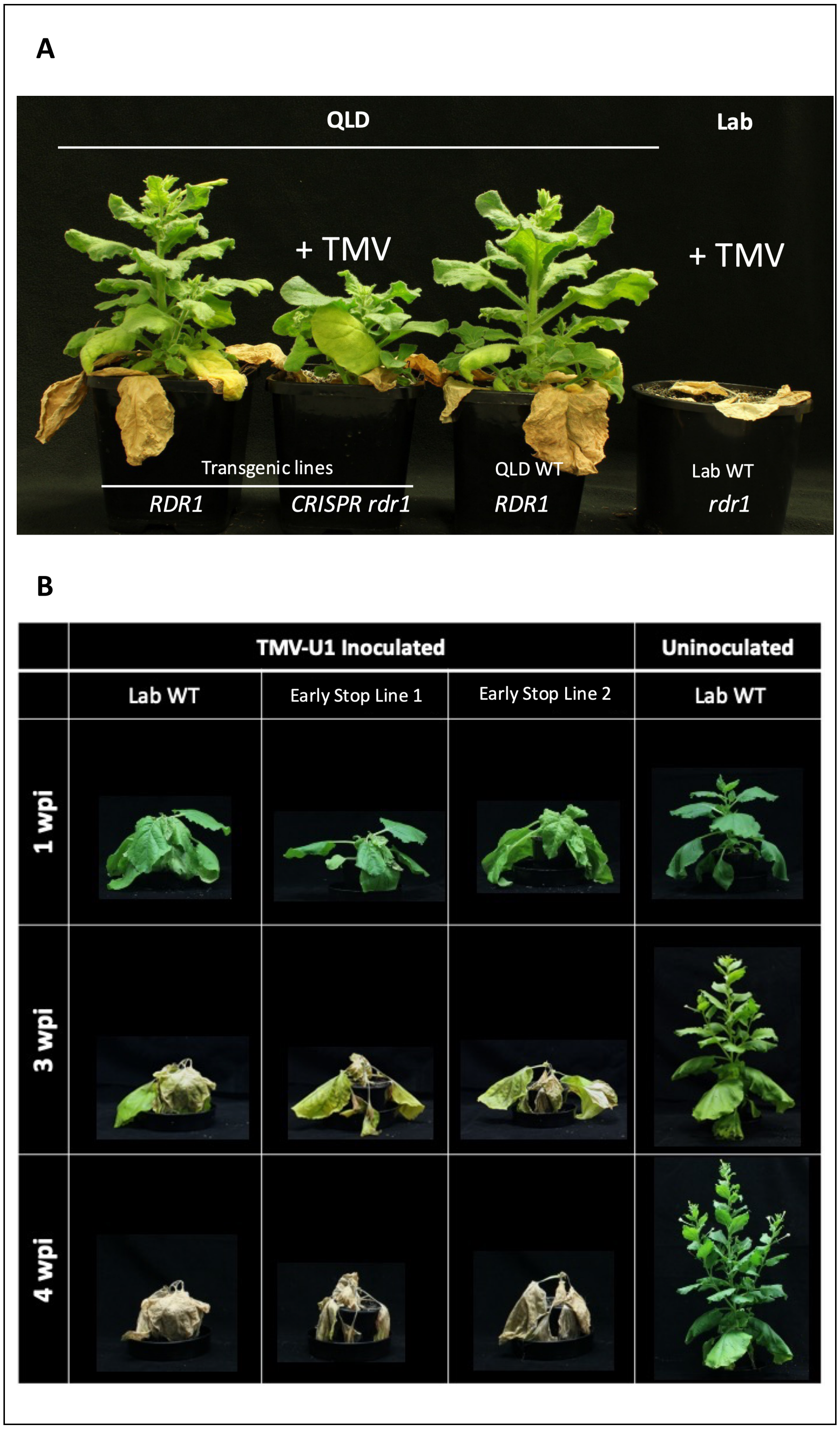

Figure S8. Effect on TMV susceptibility of editing a deletion in RDR1 in LAB and QLD.

(A) Eight-week-old non-transgenic LAB (naturally RDR1 defective) and QLD (naturally RDR1 competent) and QLD engineered, using CRISPR, to have a defective RDR1. Four-week-old plants were challenged with tobacco mosaic virus U1.

(B) One, three and four weeks after inoculation of four-week-old LAB plants. Two lines were engineered to have early stop codons to test for a dominant negative effect. The early stops did not affect the viral susceptibility suggesting that the natural 72nt insertion is not creating a dominant negative effect.

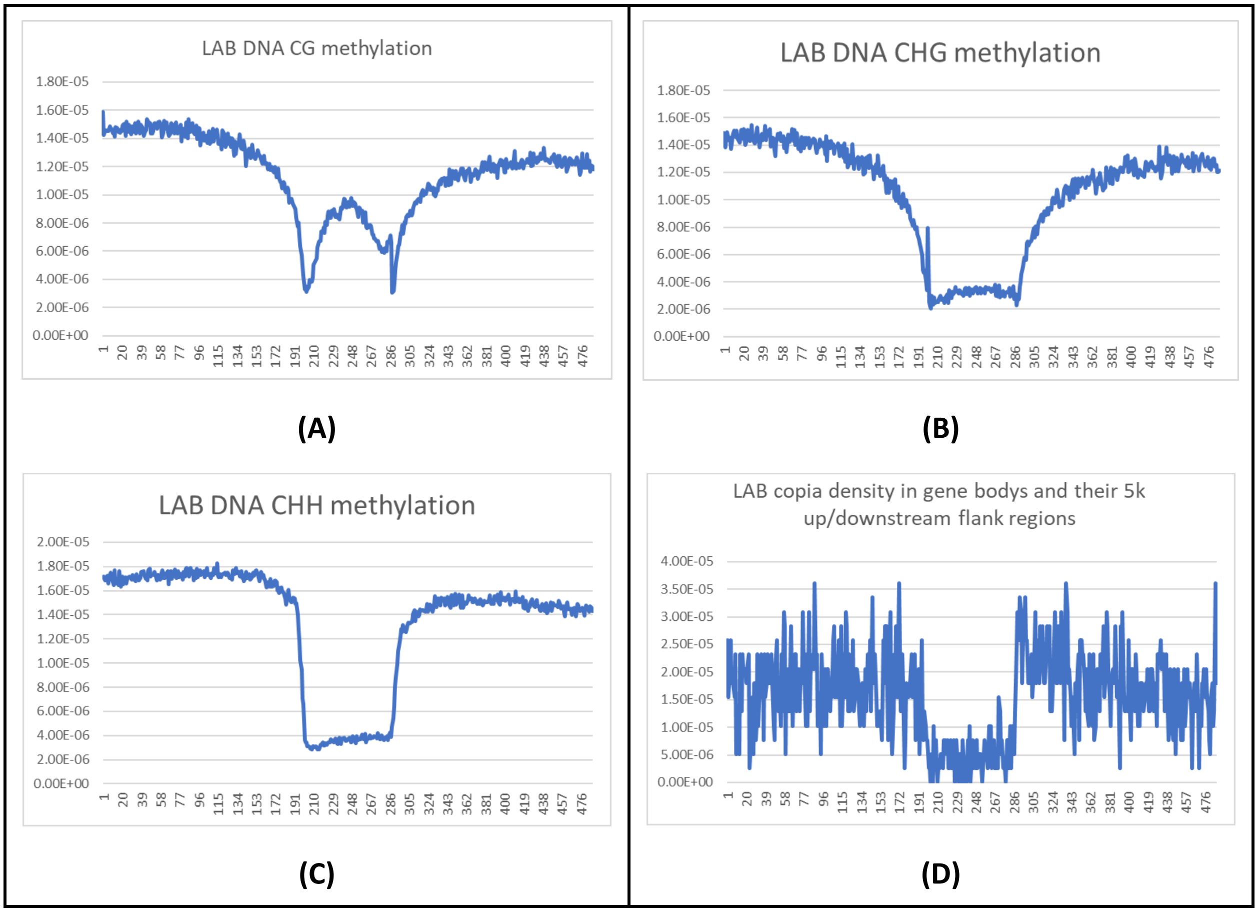

Figure S9. Copia density and DNA methylation profiles in the proximity of LAB genes. The vertical axis represents the number of Copia elements per bin of size 25bp, and the horizontal axis represents the gene region and its flanking regions. The first segment ranges from 1 to 200, representing the upstream flanking region (5,000bp/25bp). The second segment ranges from 200 to 300, representing the coding region (average gene length 2,517bp/25bp). The last segment ranges from 300 to 500, representing the downstream flanking region (5,000+5,000+2,517/25). Gene lengths have been normalised to the average gene length**.** Panels (A), (B), (C), and (D) show CG, CHG, CHH, and Copia elements, respectively.

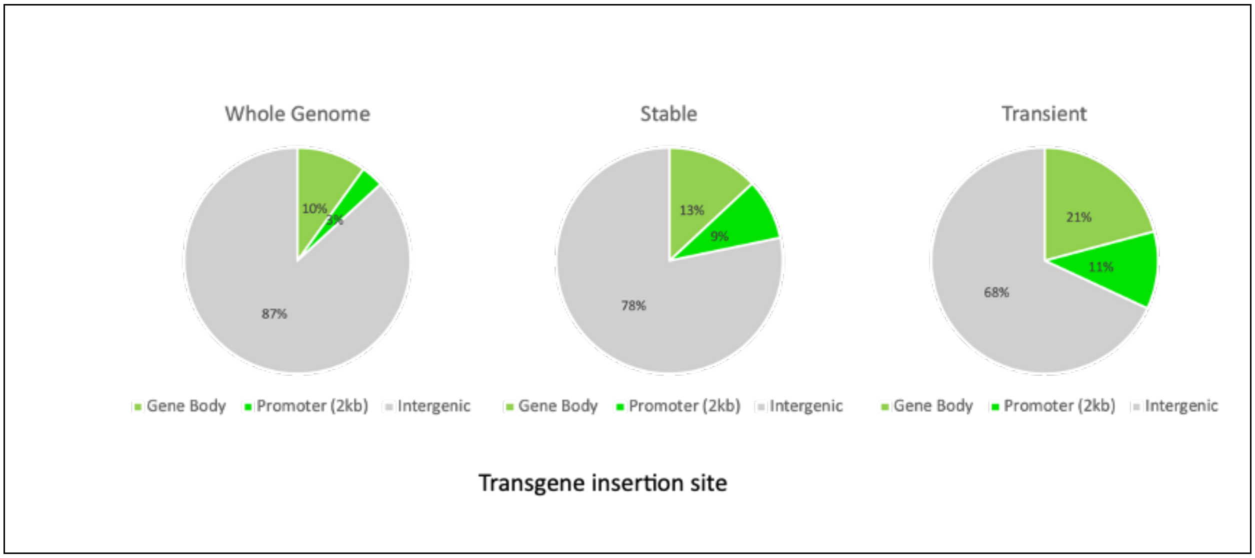

Figure S10. Association of genomic features with T-DNA genomic junctions. The portion of some genomic features: gene body, promoters (2kb upstream), and intergenic regions represented across the whole genome of *N. benthamiana* and the percentage of independent stable and transient T-DNA- insertions occurred within gene body, promoters (2kb upstream), and intergenic regions. The majority of T-DNA- insertions occurred within the intergenic regions while 22% of stable and 32% of transient T-DNA- insertions occurred within the gene body and promoter (2kb upstream) regions.

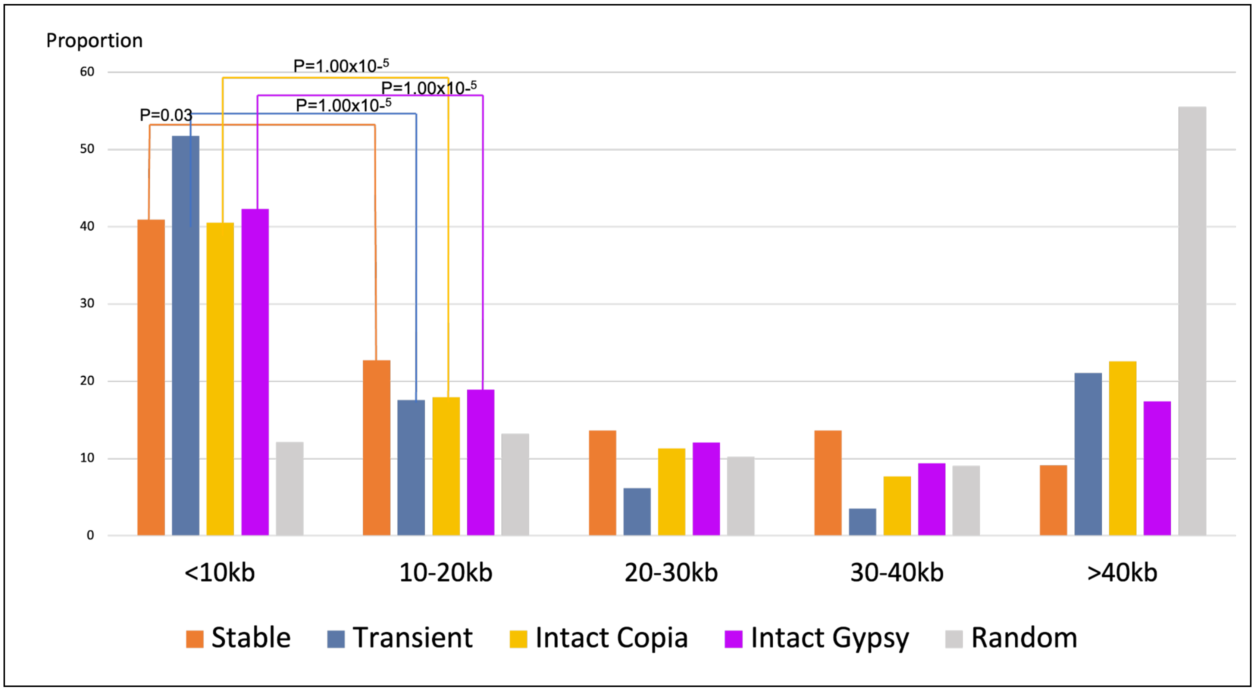

Figure S11. Distance, in 10kb categories, to the closest gene for stable and transient transgene insertion sites, intact Copia and Gypsy insertion sites, and random genomic sites. The z-score test for two population proportions was used to determine the significant difference between <10kb, 10-20kb, 20-30kb and 30-40kb intervals from all stable, transient transgene insertion sites, intact copia and gypsy insertions and randomly selected sites in the *N. benthamiana* genome. A significant difference was observed only between <10kb and 10-20kb and significant p-values are shown for each comparison.

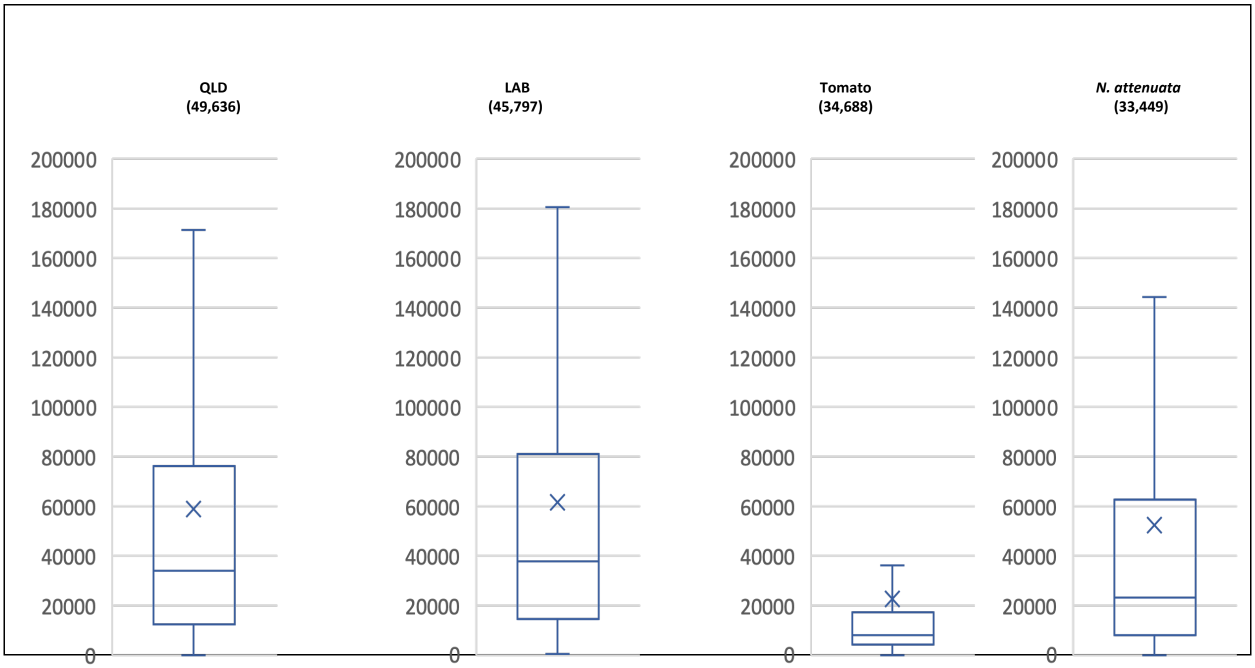

Figure S12. Box and whisker plot of average intergenic distances of LAB, QLD, tomato and *N. attenuata* genomes. The genome version used in this analysis was QLD183. LAB360, Tomato 4.1 and *N. attenuata* 2.0**.** The number of genes per genome is shown in brackets**.** Mean values are shown in each box as a cross ("X"); the line across the box indicates the median of the intergenic distance. Each box represents the range of distances that fall into the second and third quartiles. The whiskers mark the 5th and 95th percentiles. LAB and QLD have similar intergenic distances (60kbp) which are slightly larger than *N. attenuata* and considerably larger than tomato.

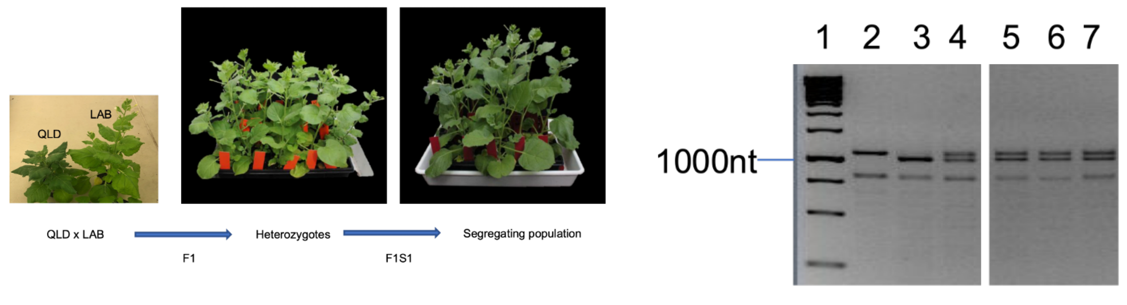

(A) **(B)**

Figure S13. Inter-fertility of LAB and QLD. (**A**) Pollen from LAB was used to fertilise emasculated flowers of QLD and vice-versa. Both directions produced capsules containing >50 seeds. Seeds were germinated and grown in soil to set F1S1 seed or backcrossed with LAB or QLD. All crosses and selfing-derived seeds gave healthy fertile plants with a variety of morphologies. (**B**) Hybridisation was confirmed by testing progeny of the initial LAB x QLD cross by PCR across the Rdr1 locus, which has a homozygous 72 bp insertion in the LAB background (n=20). Lanes 2, 3 and 4 are LAB (homozygous insertion), QLD (homozygous no insertion), and a known heterozygote, respectively. Lanes 5, 6 and 7 are samples from three plants generated in the LAB x QLD cross.
