# Supplementary material for: A multi-omic Nicotiana benthamiana resource for fundamental research and biotechnology
Source: Nat Plants. 2023 Aug 10;9(9):1558–71. doi: 10.1038/s41477-023-01489-8 (PMC10505560; doi:10.1038/s41477-023-01489-8)
Supplement: Supplementary file 2 — Reporting Summary [file 41477_2023_1489_MOESM2_ESM.pdf]

nature portfolio

Corresponding author(s): Peter Waterhouse

Last updated by author(s): Jul 4, 2023

Reporting Summary

Nature Portfolio wishes to improve the reproducibility of the work that we publish. This form provides structure for consistency and transparency in reporting. For further information on Nature Portfolio policies, see our Editorial Policies and the Editorial Policy Checklist.

Statistics

For all statistical analyses, confirm that the following items are present in the figure legend, table legend, main text, or Methods section.

n/a	Confirmed	
☐	☒	The exact sample size (*n*) for each experimental group/condition, given as a discrete number and unit of measurement
☐	☒	A statement on whether measurements were taken from distinct samples or whether the same sample was measured repeatedly
☐	☒	The statistical test(s) used AND whether they are one- or two-sided<br>*Only common tests should be described solely by name; describe more complex techniques in the Methods section.*
☒	☐	A description of all covariates tested
☒	☐	A description of any assumptions or corrections, such as tests of normality and adjustment for multiple comparisons
☐	☒	A full description of the statistical parameters including central tendency (e.g. means) or other basic estimates (e.g. regression coefficient) AND variation (e.g. standard deviation) or associated estimates of uncertainty (e.g. confidence intervals)
☐	☒	For null hypothesis testing, the test statistic (e.g. *F*, *t*, *r*) with confidence intervals, effect sizes, degrees of freedom and *P* value noted<br>*Give P values as exact values whenever suitable.*
☒	☐	For Bayesian analysis, information on the choice of priors and Markov chain Monte Carlo settings
☒	☐	For hierarchical and complex designs, identification of the appropriate level for tests and full reporting of outcomes
☒	☐	Estimates of effect sizes (e.g. Cohen's *d*, Pearson's *r*), indicating how they were calculated

Our web collection on statistics for biologists contains articles on many of the points above.

Software and code

Policy information about availability of computer code

Data collection	No software was used for data collection in this study
Data analysis	Software used: Merqury software (version 1.3), LAI sub-package of the LTR-retriever package (https://github.com/oushujun/EDTA/wiki/Calculate-LAI-from-EDTA-GFF3-files), CANU (version 1.81), SparseAssembler k-mer 77 (version 20160205), DBG2OLC (version 20160205), Racon (version 1.3.2), Juicebox (version 2.12), Citrus (https://github.com/anjiyuan/Citrus), LR_Gapcloser (version 1.1), Pilon (version 1.23), HISAT2 (version 2.1.0), Scallop (version 0.10.5), Transdecoder (https://github.com/TransDecoder/TransDecoder/), AUGUSTUS (version 3.2.3), Orthofinder (version 2.5.4), UpSetR package (version 1.4.0), EDTA pipeline (version 2.0.0) (https://github.com/oushujun/EDTA), Bowtie (version 2.0), miRPlant (version 6), bowtie2 (version 2.3.5), Picard toolkit's (https://broadinstitute.github.io/picard/) (version 2.19) MarkDuplicates SAMtools (version 1.10), SGSautoSNP pipeline (version 2.001), bowtie2 (version 2.4.2), The deepTools, (Version 3.5.1) bamCompare, Bismark program (Version 0.19.0), MCScanX toolkit OmicsBox (version 2.2.4), Muscle (version 3.8), jModeltest2 (version 2.1), MrBayes (version 3.2.6), Burrows-Wheeler (BWA-MEM) (Version 0.7), bedtools Intersect tool (version 2.30.0), RnaChipIntegrator (Version 1.1.0)

For manuscripts utilizing custom algorithms or software that are central to the research but not yet described in published literature, software must be made available to editors and reviewers. We strongly encourage code deposition in a community repository (e.g. GitHub). See the Nature Portfolio guidelines for submitting code & software for further information.

Data

Policy information about availability of data

All manuscripts must include a data availability statement. This statement should provide the following information, where applicable:

- Accession codes, unique identifiers, or web links for publicly available datasets
- A description of any restrictions on data availability
- For clinical datasets or third party data, please ensure that the statement adheres to our policy

The Nicotiana benthamiana genome and transcriptome assemblies, along with their annotations, can be accessed at https://apollo.nbenth.com/. The raw data utilized for genome assembly has been deposited in the NCBI Sequence Read Archive (SRA) under BioProject PRJNA881799. Specifically, the PacBio data for LAB and QLD can be found under the accessions SRR21820240 and SRR21820239, respectively. The HiC data for LAB and QLD are available under the accessions SRR21820238 and SRR21820237, respectively.
Databases used: KEGG (https://www.genome.jp/kegg/compound/), Metfrag (https://ipb-halle.github.io/MetFrag/projects/metfragweb/), PubChem mass databases (ST3) (https://pubchem.ncbi.nlm.nih.gov/), miRbase (release 21; https://www.mirbase.org/) and Nicotiana attenuata Data Hub (http://nadh.ice.mpg.de/NaDH/others/data)

Human research participants

Policy information about studies involving human research participants and Sex and Gender in Research.

Reporting on sex and gender	N/A
Population characteristics	N/A
Recruitment	N/A
Ethics oversight	N/A

Note that full information on the approval of the study protocol must also be provided in the manuscript.

Field-specific reporting

Please select the one below that is the best fit for your research. If you are not sure, read the appropriate sections before making your selection.

☒ Life sciences ☐ Behavioural & social sciences ☐ Ecological, evolutionary & environmental sciences

For a reference copy of the document with all sections, see nature.com/documents/nr-reporting-summary-flat.pdf

Life sciences study design

All studies must disclose on these points even when the disclosure is negative.

Sample size	No sample size calculation was performed. Sample sizes were determined according to similar studies in the field. This approach ensured that the selected sample sizes would enable confident statistical analysis while considering factors such as the cost of analysis and the sample availability.
Data exclusions	No data were excluded.
Replication	All experiments using multiple samples (eg transgene expression levels, described in extended data) had at least 3 biological replicates and 2 technical replicates. In most experiments, considerably more replicates were use and the n values are reported in the text and figures.
Randomization	For most of the experiments conducted, randomization was not necessary. However, for specific experiments such as the comparison of plant transformation and editing efficiency, RNAseq and metabolomics, ChIP-seq, whole genome bisulfite sequencing as well as transgene insertion experiments, randomization of plants was implemented in both the tissue culture and growth rooms to mitigate the impact of confounding variables and increases the validity and reliability of the experimental results.
Blinding	Since this paper primarily focuses on the assembly and analysis of the N. benthamiana genome, blinding was not applicable to this study due to the nature of the experimental design and the specific procedures involved. These processes are computational and technical in nature, involving the application of specialized algorithms, bioinformatics tools, and statistical analyses.<br>The material used for biological experiments; plant transformation and editing efficiency, RNAseq and metabolomics, ChIP-seq, whole

genome bisulfite sequencing and transgene insertions were collected from randomized plants under controlled conditions.

Reporting for specific materials, systems and methods

We require information from authors about some types of materials, experimental systems and methods used in many studies. Here, indicate whether each material, system or method listed is relevant to your study. If you are not sure if a list item applies to your research, read the appropriate section before selecting a response.

Materials & experimental systems

n/a	Involved in the study
☐	☒ Antibodies
☒	☐ Eukaryotic cell lines
☒	☐ Palaeontology and archaeology
☒	☐ Animals and other organisms
☒	☐ Clinical data
☒	☐ Dual use research of concern

Methods

n/a	Involved in the study
☐	☒ ChIP-seq
☒	☐ Flow cytometry
☒	☐ MRI-based neuroimaging

Antibodies

Antibodies used

Antibodies:
Manufacturer: Abcam
Catalog/lot number: Anti-Histone H3 (tri methyl K4) antibody - ChIP Grade (ab8580)
Used at a 1:100 dilution.

Manufacturer: Abcam
Catalog/lot number: Anti-Histone H3 (tri methyl K27) antibody - ChIP Grade (Abcam ab6002)
Used at a 1:100 dilution.

Manufacturer: Abcam
Catalog/lot number: Anti-Histone H3 (acetyl K27) antibody - ChIP Grade (ab4729)
Used at a 1:100 dilution.

Manufacturer: Diagenode
Catalog/lot number: H3K9me2 polyclonal antibody-Classic (Diagenode C15410060)
Used at a 1:100 dilution.

Validation

Anti-Histone H3 (tri methyl K4) antibody - ChIP Grade (ab8580) validated by manufacturer Abcam (Manufacturere's information available at https://www.abcam.com/products/primary-antibodies/histone-h3-tri-methyl-k4-antibody-chip-grade-ab8580.html?productWallTab=ShowAll)

Anti-Histone H3 (tri methyl K27) antibody - ChIP Grade (Abcam ab6002) validated by manufacturer Abcam (Manufacturer's information available at
https://www.abcam.com/products/primary-antibodies/histone-h3-tri-methyl-k27-antibody-mabcam-6002-chip-grade-ab6002.html?productWallTab=ShowAll)

Anti-Histone H3 (acetyl K27) antibody - ChIP Grade (ab4729) validated by manufacturer Abcam (Manufacturer's information available at https://www.abcam.com/products/primary-antibodies/histone-h3-acetyl-k27-antibody-chip-grade-ab4729.html)

H3K9me2 polyclonal antibody-Classic (Diagenode C15410060) validated by manufacturer Diagenode (Manufacturer's information available at https://www.diagenode.com/en/p/h3k9me2-polyclonal-antibody-classic-50-ug-44-ul#)

ChIP-seq

Data deposition

☒ Confirm that both raw and final processed data have been deposited in a public database such as GEO.

☐ Confirm that you have deposited or provided access to graph files (e.g. BED files) for the called peaks.

Data access links
May remain private before publication.

Web Apollo platform https://www.nbenth.com/
LAB v3.6 and QLD v 1.83
BioProject PRJNA881799. http://www.ncbi.nlm.nih.gov/bioproject/881799

Files in database submission	Files available from BioProject ID: PRJNA881799 http://www.ncbi.nlm.nih.gov/bioproject/881799
Genome browser session (e.g. UCSC)	Web Apollo platform https://www.nbenth.com/

Methodology

Replicates

The genome-wide histone modification landscapes of LAB and QLD. Libraries were determined using two replicates per histone modification and control input

Sequencing depth

Sample	Number of total Reads (paired)	Number of mapped reads (paired)
L_H3K27acR1	87,766,459	72,187,913
L_H3K27acR2	111,137,613	86,887,386
L_H3K4me3R1	101,420,324	80,740,720
L_H3K4me3R2	103,062,025	87,231,698
L_H3K27me3R1	101,644,913	49,226,631
L_H3K27me3R2	87,125,829	71,617,431
L_H3K9me2R1	32,710,161	31,143,344
L_H3K9me2R2	349,788,182	299,442,004
L_Input1	121,539,580	72,680,669
L_Input2	120,448,239	60,356,613
L_Input3	174,002,911	129,771,371
L_Input4	121,031,125	119,748,195
Q_H3K27acR1	106,529,454	86,214,287
Q_H3K27acR2	91,504,824	75,226,116
Q_H3K4me3R1	98,117,437	68,947,123
Q_H3K4me3R2	87,068,856	67,800,518
Q_H3K27me3R1	97,125,964	39,287,452
Q_H3K27me3R2	117,728,178	79,254,609
Q_H3K9me2R1	235,913,497	211,755,955
Q_H3K9me2R2	27,665,306	25,900,259
Q_Input1	124,174,832	64,099,048
Q_Input2	106,428,021	60,483,044
Q_Input3	107,547,910	101,417,679
Q_Input4	125,052,817	118,062,365

Alignments with MAPQ of < 40 were discarded prior to downstream analyses to ensure homeolog specificity and accuracy in polyploid N. benthamiana genome.

Antibodies

Antibodies against two active histone marks, anti-histone-H3-tri-methyl-K4 (Abcam ab8580) and anti-histone-H3-acetyl-K27 (Abcam ab4729), and two repressive histone marks, anti-histone-H3-tri-methyl-K27 (Abcam ab6002) and anti-histone-H3-di-methyl-K9 (Diagenode C15410060)

Peak calling parameters

No peak calling was used in this study

Data quality

ChIP-seq reads generated from the experiment were mapped to the N.benthamiana (LAB and QLD) genomes. Only uniquely mapped reads after removing duplicated reads (MAPQ>40) were used for downstream analysis. The pairwise correlation (spearman correlation) between replicates (>0.80) was computed using plotCorrelation. The signal strength of ChIP samples was confirmed using plotFingerprint and ChIP enrichment over the background was visualised on IGV (https://software.broadinstitute.org/software/igv/) browser.

Software

deepTools2 and bamCompare.
Ramírez, F. et al. deepTools2: a next generation web server for deep-sequencing data analysis. Nucleic Acids Res. 44, W160–5 (2016).
