# Supplementary figures and images for: A multi-omic Nicotiana benthamiana resource for fundamental research and biotechnology
Source: Nat Plants. 2023 Aug 10;9(9):1558–71. doi: 10.1038/s41477-023-01489-8 (PMC10505560; doi:10.1038/s41477-023-01489-8)

QLD GEL

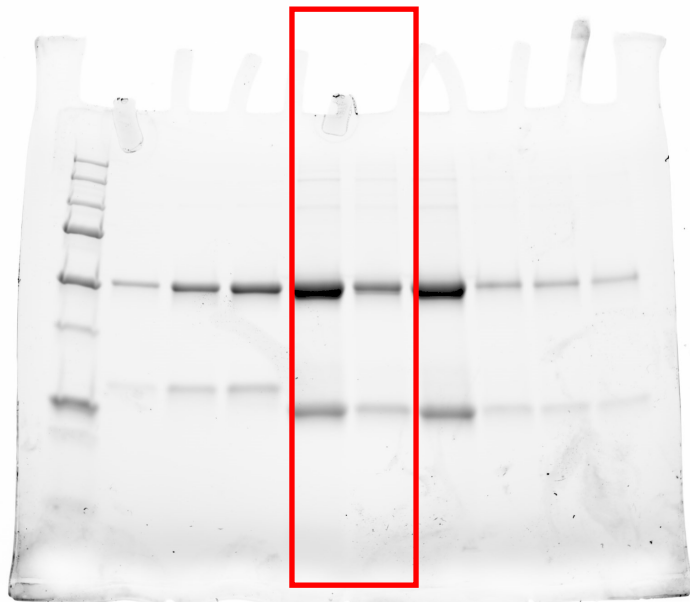

LAB GEL

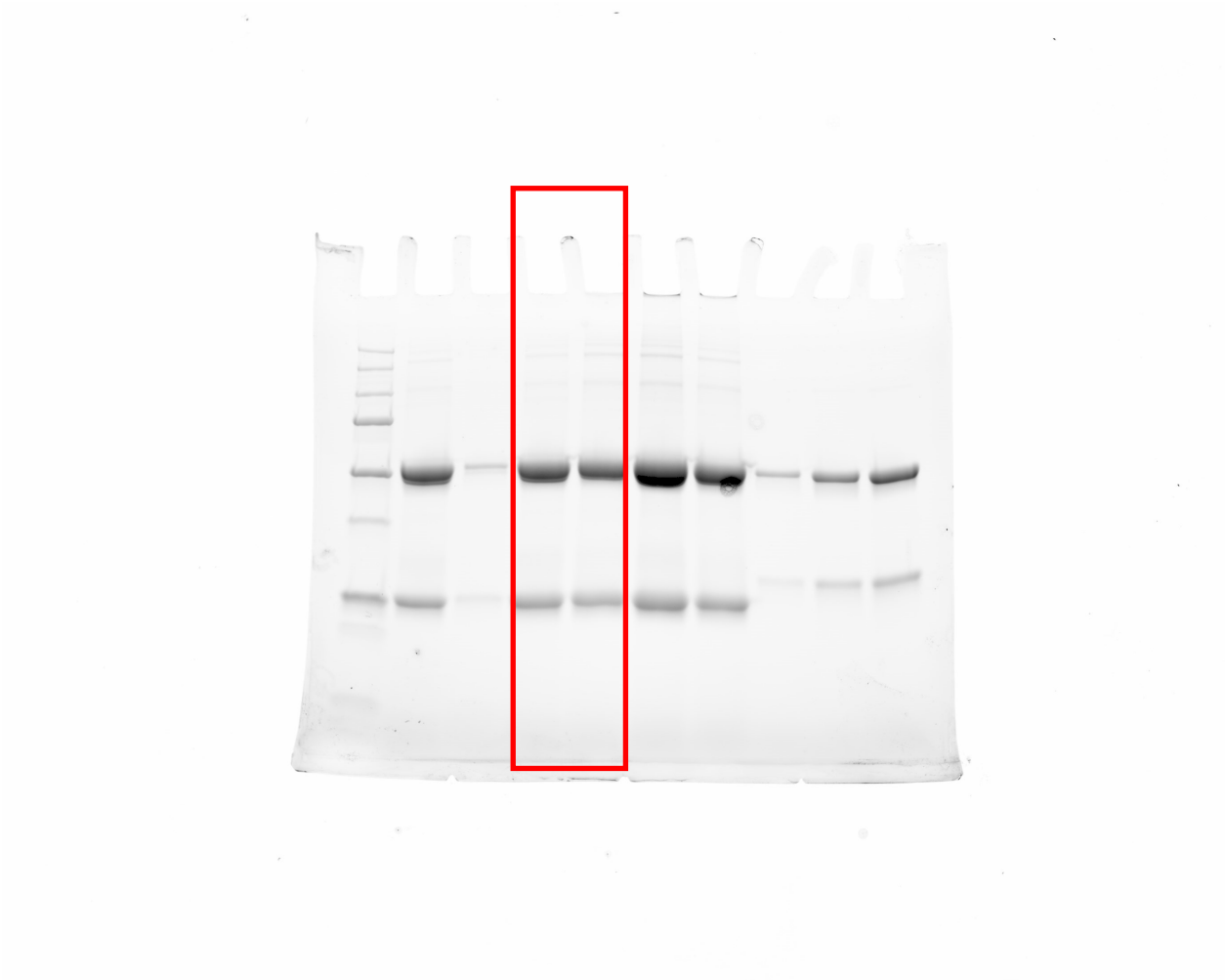

Supplement: Supplementary file 4 — Unprocessed SDS–PAGE. [file 41477_2023_1489_MOESM4_ESM.pdf]
